# Understanding the quality of ethnicity data recorded in health-related administrative data sources compared with Census 2021 in England

**DOI:** 10.1371/journal.pmed.1004507

**Published:** 2025-02-26

**Authors:** Cameron Razieh, Bethan Powell, Rosemary Drummond, Isobel L. Ward, Jasper Morgan, Myer Glickman, Chris White, Francesco Zaccardi, Jonathan Hope, Veena Raleigh, Ashley Akbari, Nazrul Islam, Thomas Yates, Lisa Murphy, Bilal A. Mateen, Kamlesh Khunti, Vahe Nafilyan

**Affiliations:** 1 Office for National Statistics, Newport, United Kingdom; 2 Leicester Real World Evidence Unit, Diabetes Research Centre, College of Life Sciences, University of Leicester, Leicester, United Kingdom; 3 Diabetes Research Centre, College of Life Sciences, University of Leicester, Leicester General Hospital, Leicester, United Kingdom; 4 NHS England, 7 and 8 Wellington Place, Leeds, United Kingdom; 5 King’s Fund, London, United Kingdom; 6 Nuffield Trust, London, United Kingdom; 7 Population Data Science, Faculty of Medicine, Health & Life Science, Swansea University, Swansea, United Kingdom; 8 Primary Care Research Centre, University of Southampton, Southampton, United Kingdom; 9 NIHR Leicester Biomedical Research Centre, Diabetes Research Centre, College of Life Sciences, University of Leicester, Leicester, United Kingdom; 10 Wellcome Trust, London, United Kingdom; 11 PATH, Seattle, Washington, United States of America; 12 University College London, Institute of Health Informatics, London, United Kingdom; 13 NIHR Applied Research Collaboration–East Midlands (ARC-EM), Leicester General Hospital, Leicester, United Kingdom; Washington University School of Medicine, UNITED STATES OF AMERICA

## Abstract

**Background:**

Electronic health records (EHRs) are increasingly used to investigate health inequalities across ethnic groups. While there are some studies showing that the recording of ethnicity in EHR is imperfect, there is no robust evidence on the accuracy between the ethnicity information recorded in various real-world sources and census data.

**Methods and findings:**

We linked primary and secondary care NHS England data sources with Census 2021 data and compared individual-level agreement of ethnicity recording in General Practice Extraction Service (GPES) Data for Pandemic Planning and Research (GDPPR), Hospital Episode Statistics (HES), Ethnic Category Information Asset (ECIA), and Talking Therapies for anxiety and depression (TT) with ethnicity reported in the census. Census ethnicity is self-reported and, therefore, regarded as the most reliable population-level source of ethnicity recording. We further assessed the impact of multiple approaches to assigning a person an ethnic category. The number of people that could be linked to census from ECIA, GDPPR, HES, and TT were 47.4m, 43.5m, 47.8m, and 6.3m, respectively. Across all 4 data sources, the White British category had the highest level of agreement with census (≥96%), followed by the Bangladeshi category (≥93%). Levels of agreement for Pakistani, Indian, and Chinese categories were ≥87%, ≥83%, and ≥80% across all sources. Agreement was lower for Mixed (≤75%) and Other (≤71%) categories across all data sources. The categories with the lowest agreement were Gypsy or Irish Traveller (≤6%), Other Black (≤19%), and Any Other Ethnic Group (≤25%) categories.

**Conclusions:**

Certain ethnic categories across all data sources have high discordance with census ethnic categories. These differences may lead to biased estimates of differences in health outcomes between ethnic groups, a critical data point used when making health policy and planning decisions.

## Abstract 2

Cameron Razieh and colleagues undertake a comparison of ethnicity data found in national health records in England with information provided by individuals in England’s Census 2021.

## Author summary

**Why was this study done?:**

- Collecting high-quality ethnicity data within administrative data sources has become a priority to governments, data providers, and the public over recent years. There is limited research investigating the quality of the recording of ethnicity across different health administrative data sources and in the limited available, it indicates that missingness is relatively high and consistency across sources varies. If ethnicity data across different health administrative data sources is inconsistent or misclassified, analyses based on different data sources may therefore lead to inconsistent and biased estimates, which could create confused messaging and flawed policy formation.

**What did the researchers do and find?:**

- This study applied and compared multiple approaches to assigning an ethnic category from episode-level records in several health administrative data sources and compared the individual-level assigned ethnic category with that collected in Census 2021, a source considered to contain high-quality ethnicity data for the whole population of England. The health administrative data sources compared to Census 2021 were: General Practice Extraction Service (GPES) Data for Pandemic Planning and Research (GDPPR); Hospital Episode Statistics (HES); NHS England’s (NHSE) Ethnic Category Information Asset (ECIA); and NHSEs Talking Therapies, for anxiety and depression (TT). The number of people that could be linked to census from ECIA, GDPPR, HES, and TT were 47.4m, 43.5m, 47.8m, and 6.3m, respectively. Across all data sources, agreement was lower for Mixed (≤75%) and Other (≤71%) categories across all data sources. The categories with the lowest agreement were Gypsy or Irish Traveller category (≤6%), Other Black (≤19%), and Any Other Ethnic Group (≤25%).

**What do these findings mean?:**

- Improving the quality of ethnicity data within health administrative data sources requires a coordinated approach from many different organisations and includes targeting a standardised definition on the term ethnicity, and standardising the methods that ethnicity data is collected, recorded, and processed. Study limitations include: the exclusion of individuals who did not take part in the census; linkage rates vary between ethnic groups; and the lack of information available on the context where and how the ethnicity information was collected within the health administrative data sources.

## Introduction

Collecting high-quality ethnicity data within administrative data sources has become a priority to governments, data providers, and the public over recent years. Electronic health records (EHRs) can be defined as the systematic collection of patients health information stored in a digital format. EHRs contain information including but not limited to demographic, diagnosis, and medication information for patients and are designed to be accessed and shared across healthcare settings, with the data being collected from primary, secondary, and other healthcare settings when a patient has interacted with the healthcare service. The recording of ethnicity in healthcare environments vary. Primary care recording varies, with most collecting self-report at registration, but an individual can ignore the question without penalty [1]. Further, in both primary and secondary care environments, ethnicity can be recorded by clinicians or administrative staff with some assumptions, with data often carried forward from previous records that were also assumed [2]. EHRs have increasingly been used to publish statistics and analysis on ethnic health inequalities. This was apparent during the COVID-19 pandemic, when people from minority ethnic groups were found to be at higher COVID-19 mortality risk [3–5]. The limited research on the quality of the recording of ethnicity across different EHRs indicates that missingness is relatively high and consistency across sources varies [6–8]. Analyses based on different data sources may, therefore, lead to inconsistent and biased estimates, which could create confused messaging and flawed policy formation.

This study builds upon previous work examining ethnicity coding between health administrative data sources [6,7,9–11]. These previous studies highlight issues with the quality of ethnicity information collected within the National Health Service (NHS). These quality issues are set in the context that health administrative data are increasingly being used to produce statistics and research. However, statistical analysis was not the initial purpose of health administrative data sets and is not their primary function. Health administrative data can be used for statistical analysis successfully, but to do so there is a need to understand their limitations. Furthermore, there is a lack of evidence assessing the quality of ethnicity data within primary care records in England to a “gold standard” comparator, using patient-level records. However, previous evidence has compared the aggregated ethnic breakdown between 2011 Census and Clinical Practice Research Datalink (CPRD) data [12,13], with the studies reporting that the ethnic breakdown between 2011 Census and CPRD were broadly similar. Further analysis examining the agreement between CPRD and HES using 5-category ethnic groups [13] and analysis examing the quality of ethnicity coding in Scottish health records compared with the 2011 Scottish Census has also been undertaken [8].

The aims of this study are to apply and compare multiple approaches to assigning an ethnic category from episode-level records in several health administrative data sources and compare the individual-level assigned ethnic category with that collected in Census 2021, a source considered to contain high-quality ethnicity data for the whole population of England [14]. The data is noted to be high quality because we can generally be confident the ethnicity data in census are self-reported and engagement is mandatory [15]. It is noted however that self-reported ethnicity and mandatory engagement may not always guarantee high-quality ethnicity data. Whereas, there is less certainty that ethnicity recordings within primary and hospital healthcare environments are self-reported [2].

## Methods

### Data sources and study population

This analysis compared the anonymised individual-level recorded ethnicity of patients in England in 4 health administrative data sources with their recorded self-assigned ethnicity in Census 2021. The 4 health administrative data sources were:

- General Practice Extraction Service (GPES) Data for Pandemic Planning and Research (GDPPR), which contains information on all active patients registered at a GP practice in England on 1 November 2019 [16];
- Hospital Episode Statistics (HES), a database containing details of all attendances at NHS hospitals in England, which is made up of three sub-data sets (Accident and Emergency (A&E), which was superseded by the Emergency Care Dataset (ECDS) in April 2020; Admitted Patient Care (APC); and Outpatients (OP)) [17];
- NHS England’s (NHSE) Ethnic Category Information Asset (ECIA)—this source combines ethnicity data from GDPPR and HES making it the most complete NHS source of ethnicity information for England [18]; and
- NHSEs Talking Therapies, for anxiety and depression (TT), formerly Improving Access to Psychological Therapies (IAPT), a data set that was developed to monitor and evaluate an NHSE programme aimed at improving the delivery of, and access to, evidence-based, psychological therapies for adults with depression and anxiety disorders [19]. This data set is therefore smaller than the rest given that not all people receive these therapies.

Census 2021 data were used as a “gold standard” comparator as ethnicity data in census are self-reported and engagement is mandatory [15]. Further, censuses are designed with user experience in mind to ensure that individuals have the requisite information to make an appropriate selection. It is noted however that these factors may not always guarantee high-quality ethnicity data. Though self-reported data collection for ethnicity is recommended [15].

### Data linkage

To enable comparisons of ethnicity recorded in each health administrative source with the Census 2021, people enumerated in Census 2021 were linked securely to the NHS Personal Demographics Service (PDS) to obtain their NHS number (with 95.75% of persons in the census probabilistically and deterministically matched to persons in the PDS) [20]. Our Census 2021 study population included 55.1 million people enumerated in England and Wales for whom we could obtain an NHS number. We then excluded individuals who were resident in Wales at the time of census (2.8 million), those who had not answered the ethnicity question within Census 2021 (0.5 million), and those who were not usual residents in England (0.4 million). Therefore, a total of 51.3 million individuals from England were included in our analysis, covering 90.8% of the population of England on Census Day 2021, which was estimated to be 56.5 million [21].

**Fig 1:**
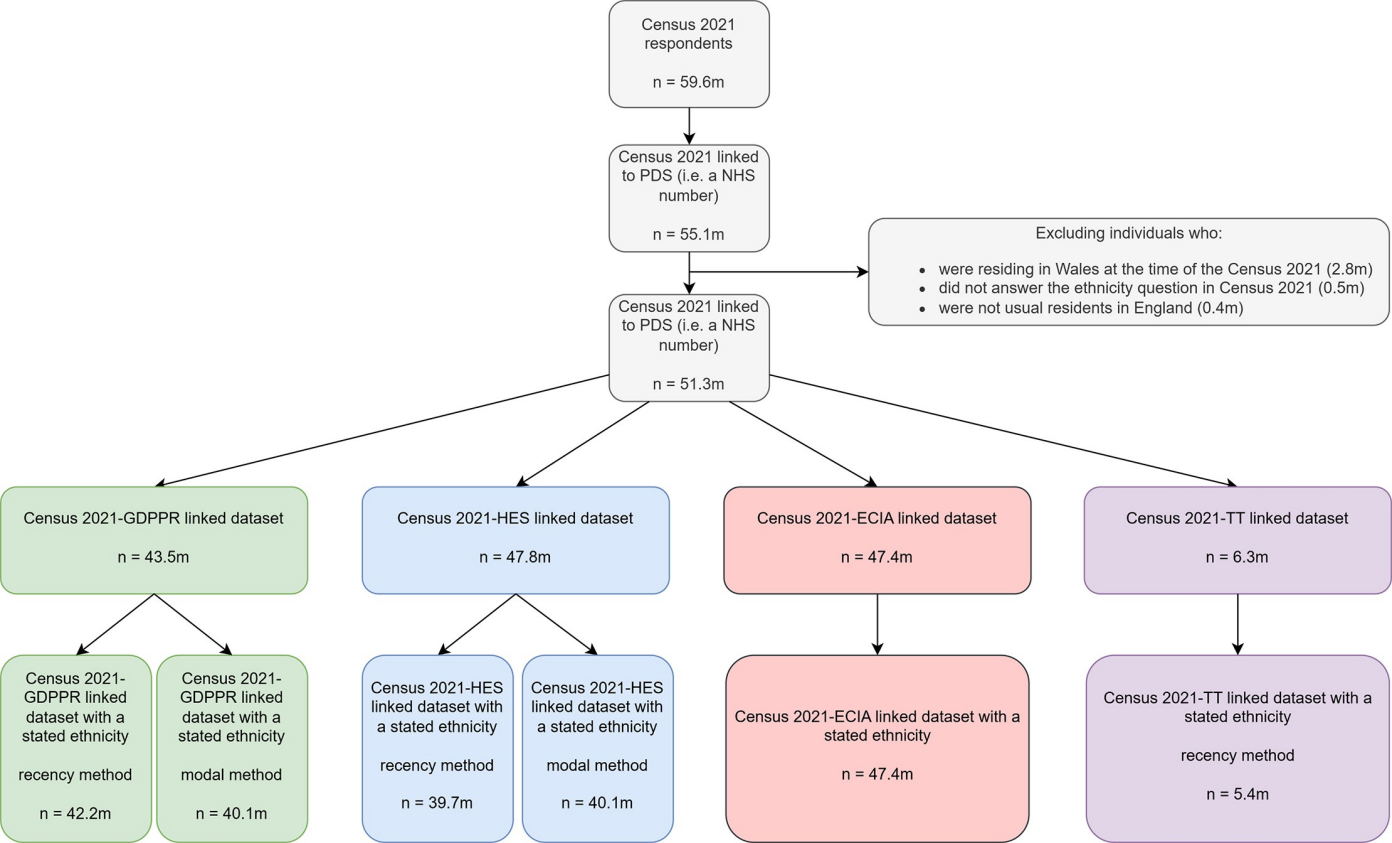
Flow chart of inclusion. For GDPPR, HES, and TT data sources, these data refer to when the Unknown only reallocation methodology has been applied. ECIA, Ethnic Category Information Asset; GDPPR, GPES Data for Pandemic Planning and Research; HES, Hospital Episode Statistics; TT, Talking Therapies.

Individuals with available ethnicity data from each health data source were then linked to census using NHS number. The count of people in the each of the linked data sets presented in our analyses are reported in **Fig 1** and **S1 Table**. For GDPPR, HES, and TT, we included all available longitudinal ethnicity records recorded up to and including 29 January 2022 (the most recent date within ECIA).

### Ethnicity definitions within each data source

Ethnic categories vary across data sources and the wording of categories also varies, even when they align across data sources [11] (**S2 Table**). Census 2021 includes 19 ethnic categories, including a newly implemented Roma category; GDPPR and ECIA both have 18 ethnic categories (GDPPR and ECIA categories are based on the 2011 Census) [22]. By contrast, TT and HES data only contain 16 ethnic categories [23]; they do not include “White: Gypsy or Irish Traveller” or “Other ethnic group: Arab” categories. The HES categories were updated in April 2001 to represent the ethnic categories as defined in the 2001 Census. In the 2011 Census, the Chinese ethnic category moved from the “Other” ethnic category to the “Asian” ethnic category, and new groups for “Gypsy or Irish Traveller” and “Arab” were added [22]. In all health administrative data sources, the Chinese ethnic category is still within the “Other” ethnic group. Both 18- and 5-category ethnic groups were used in this analysis (**S3 Table**).

### Handling multiple ethnicity records per person

GDPPR, HES, and TT contain information about all interactions a patient has with the relevant health service, so generally contain multiple records per patient. Within these data sources, some individuals have multiple recorded ethnicities within the same data source at different episodes: A set of rules was therefore implemented to select a single ethnicity per person for comparison with Census 2021. In contrast, the ECIA contains a single ethnicity per person, based on the most recent ethnicity recorded in either GDPPR or HES. Full details of the methodology used to determine this have been published by NHSE [24].

We applied 2 methods to derive an individual’s ethnicity within GDPPR, HES, and TT sources: the most common (modal) and most recent (recency) ethnicity recorded for each person.

For GDPPR data, we derived the most recent ethnicity recording by taking it from either the GP-Journal (SNOMED codes) or GP-Patient (ETHNIC column) tables; priority was given to the GP-Journal table recording in instances of conflict in recoding on the same most recent date between sources. Priority was given to the GP-Journal table because the data is more granular and because of previous NHSE methodology [24]. Where conflicts on the same most recent date within the same table persisted (i.e., GP-Journal or GP-Patient tables), the ethnicity recording was classified as “Unresolved.”

To derive the most common ethnicity recording within GDPPR, we firstly determined the most frequently recorded ethnicity within the GP-Journal table. All ethnicity SNOMED codes for a person were identified and then the most frequently recorded ethnic category was identified. SNOMED codes are the clinical coding standards used with GP records [25]. Where a person did not have an ethnicity recording in the GP-Journal table (i.e., had no available SNOMED codes providing ethnicity information), we calculated the most frequently recorded ethnicity within the GP-Patient table (i.e., NHSE 18-category ethnicity codes). If a person had 2 or more most frequently recorded ethnicities, their ethnicity recording was marked as “Unresolved.”

For HES data, if there were multiple ethnicities recorded on the same most recent date for the recency definition, the records were prioritised according to the sub-data set for HES: in order HES-APC, HES-A&E/ECDS, HES-OP [12]. If conflicts still existed on the same date within the same sub-data set, the ethnicity was classified as “Unresolved.” For the modal definition in HES, no priority was applied and the most frequently selected ethnic category was selected across all sub-data sets. If a person had 2 or more most frequently recorded ethnicities, their ethnicity recording was marked as “Unresolved.”

Because of the way the extract of TT data available to us was structured and processed, no modal definition was possible. Our extract of TT data was pre-processed and assigns the most recent ethnicity recording per year and per supplier. To derive the most recent ethnicity recording within TT, we selected the most recent ethnicity recording from the most recent supplier in the most recent year available. If there were 2 or more different ethnicity recordings on the same most recent date, they were classified as “Unresolved.” The categories Data Not Recorded and Value Outside of National Code were treated like the Not Known category.

### Reallocating ethnicity records

Once a single ethnicity recording was derived for each person in GDPPR, HES, and TT using recency and modal methododologies, a further set of reallocation rules were implemented, whereby certain ethnic categories were reallocated if alternative ethnic categories were available within their records, even if these records were older or less frequent. Reallocation was applied if a person’s most recent or frequent ethnic category was Not Known, Not Stated, or Any Other Ethnic Group. This was undertaken to test whether reallocating certain ethnic categories improved agreement with Census 2021 data. The Not Known and Not Stated categories were chosen for reallocation because they effectively represent missing entries. Any Other Ethnic Group was reallocated because of evidence suggesting there is likely over-coding of this ethnic group [7]. Where an individual had only one or more recording of the same ethnicity, their ethnicity recording was not reallocated. We applied the reallocation methodologies within the entire time series of data up to 29 January 2022 for GDPPR, HES, and TT data sources. The order of the ethnic categories that were sequentially reallocated was:

1. Not Known
2. Not Known; Any Other Ethnic Group*
3. Not Known; Any Other Ethnic Group; Not Stated**

* Where a person only had Not Known and Any Other Ethnic Group categories recorded, Any Other Ethnic Group was given priority and chosen as the reallocation destination.

** Where a person only had Not Known and/or Not Stated and Any Other Ethnic Group categories recorded, Any Other Ethnic Group was given priority and chosen as the reallocation destination.

### Statistical analysis

To explore the consistency of ethnicity information across data sources, we produced 18- and 5-category ethnic category crosstabulations of Census 2021 with each health administrative data source. This enabled the examination of the distribution between each ethnic category assigned in ECIA, GDPPR, HES, and TT sources, and the ethnic category an individual was assigned in Census 2021, as counts and proportions. Statistical disclosure control rules were applied to protect personal information: all values in crosstabulations were suppressed for counts less than 10. Counts of 10 or above were rounded to the nearest 5. Percentage agreement was calculated using rounded and suppressed values.

To summarise the information contained in the crosstabulations, we presented the agreement for each health data source compared with Census 2021. Overall agreement between each respective data source with census and individual agreement per ethnic group within source were calculated. For each person, the ethnic category recorded in census and each respective data source were compared and classified as: 1, if the recorded ethnicities were the same; 0, if they were different. Where the ethnic categories used in the health administrative sources data did not exactly match with the Census 2021 categories, ethnic categories were matched with the most aligned Census 2021 ethnicity category (**S2 Table**). Only those with a stated ethnicity category in both data sources were included in the agreement calculations; the Not Stated, Not Known, and Unresolved categories were not included in agreement calculations because these categories or equivalent categories were not available within Census 2021. Arab and Traveller ethnic categories are not available within HES and TT, and therefore no agreement was calculated for these categories in HES and TT. The statistical codes used to derive ethnicity are publicly available on GitHub (ONS-Health-modelling-hub). We provide worked examples of the agreement calculation in our corresponding ONS release [26].

We also calculated sensitivity and positive predictive value (PPV) for all comparisons in line with previous validation studies; sensitivity gave the percentage of individuals with a particular ethnicity recording in Census 2021 who had a corresponding recording in each health administrative data source, while PPV gave the percentage of individuals with a particular ethnicity recording in each health administrative data source who had a corresponding ethnicity recording in the Census 2021 [8,27].

We conducted 2 sensitivity analyses where we restricted the back series of ethnicity data to 1 April 2015 (aligning to the first date within our extract of ECIA) and restricted the population to only those who had a stated ethnic category in each of the GDPPR, HES, and Census 2021 datasets. We did this to assess the extent to which agreement was affected by differences in coverage and populations between GDPPR and HES.

This study is reported as per the Strengthening the Reporting of Observational Studies in Epidemiology (STROBE) guideline (**S1 STROBE Checklist**).

## Results

### Ethnicity breakdown per data source

The number of people included who were linked to Census 2021 from ECIA, GDPPR, HES, and TT were 47.4, 43.5, 47.8, and 6.3 million, respectively (**Fig 1**). In the data sets before any reallocation methodologies were applied, the White British category was the largest ethnic category across all data sources, with the White Other category being the second largest category within the ECIA, GDPPR, and TT (**Table 1**). The percentage of people with a Not Stated or Not Known category differed across data sources: GDPPR had the lowest percentage of people assigned the Not Stated category (modal: 2.2%; recency: 2.6%), with a neglible number of people assigned a Not Known category. Within HES, the percentage of people assigned Not Stated or Not Known categories were 9% (modal) and 12% (recency) and 10% (modal) and 9.9% (recency), respectively. The percentage of people assigned a Not Stated or Not Known category in TT was 5.7% and 7.6%, respectively. TT also had Data Not Recorded and Value Outside of National Code categories, which totalled 1.4% of people within TT being assigned these categories. Compared with recency, the modal methodology to assign a person an ethnic category resulted in more Unresolved cases (i.e., conflicts in recording) in both GDPPR and HES. All other ethnic categories were ≤3.2% of the overall percentage of individuals across all data sources.

**Table 1:** 18-category ethnic breakdown per data source.

	Data source						
Ethnic Category	Census 2021 Millions *n* (%)	ECIA Millions *n* (%)	GDPPR-modal Millions *n* (%)	GDPPR-recency Millions *n* (%)	HES-modal Millions *n* (%)	HES-recency Millions *n* (%)	NHS TT-recency Millions *n* (%)
**White British**	38.5 (75)	35.1 (74.1)	30.2 (69.5)	31 (71.1)	28.5 (59.7)	28.5 (59.7)	4.4 (69.7)
**Other White**	3.1 (6)	4 (8.5)	3.3 (7.6)	3.9 (9)	2.2 (4.7)	2.3 (4.9)	0.2 (3.8)
**Indian**	1.6 (3.2)	1.4 (3.1)	1.3 (3.1)	1.4 (3.2)	1 (2)	0.9 (2)	0.1 (1.8)
**Pakistani**	1.4 (2.7)	1.2 (2.6)	1.1 (2.6)	1.2 (2.7)	0.9 (1.9)	0.9 (1.9)	0.1 (1.4)
**Black African**	1.2 (2.3)	0.9 (1.9)	0.9 (2)	0.8 (1.9)	0.6 (1.4)	0.7 (1.4)	0.1 (1)
**Other Asian**	0.8 (1.6)	0.9 (1.9)	0.7 (1.6)	0.8 (1.8)	0.6 (1.3)	0.7 (1.4)	0.1 (0.9)
**Any Other Ethnic Group**	0.8 (1.5)	1 (2.2)	0.5 (1.1)	0.7 (1.5)	0.9 (1.9)	1.1 (2.4)	0.1 (1.2)
**Bangladeshi**	0.5 (1.1)	0.5 (1)	0.4 (1)	0.4 (1)	0.3 (0.7)	0.3 (0.7)	0 (0.5)
**Black Caribbean**	0.5 (1)	0.4 (0.8)	0.3 (0.7)	0.3 (0.7)	0.3 (0.6)	0.3 (0.6)	0.1 (0.5)
**Irish**	0.4 (0.9)	0.3 (0.6)	0.2 (0.5)	0.3 (0.6)	0.2 (0.4)	0.2 (0.4)	0 (0.7)
**White and Black Caribbean**	0.4 (0.8)	0.2 (0.5)	0.2 (0.4)	0.2 (0.5)	0.2 (0.3)	0.2 (0.3)	0 (0.7)
**White and Asian**	0.4 (0.8)	0.2 (0.4)	0.1 (0.3)	0.2 (0.4)	0.1 (0.3)	0.1 (0.3)	0 (0.4)
**Other Mixed**	0.4 (0.8)	0.4 (0.8)	0.2 (0.5)	0.3 (0.7)	0.3 (0.6)	0.3 (0.7)	0 (0.7)
**Chinese**	0.3 (0.7)	0.3 (0.6)	0.2 (0.6)	0.3 (0.6)	0.2 (0.3)	0.2 (0.3)	0 (0.2)
**Arab**	0.3 (0.5)	0 (0)	0 (0.1)	0 (0.1)	No data	No data	No data
**Other Black**	0.2 (0.5)	0.4 (0.7)	0.2 (0.4)	0.3 (0.7)	0.2 (0.5)	0.3 (0.6)	0 (0.3)
**White and Black African**	0.2 (0.4)	0.2 (0.3)	0.1 (0.2)	0.2 (0.4)	0.1 (0.2)	0.1 (0.2)	0 (0.2)
**Roma**	0.1 (0.2)	No data	No data	No data	No data	No data	No data
**Gypsy or Irish Traveller**	0.1 (0.1)	0 (0)	0 (0)	0 (0.1)	No data	No data	No data
**Not Stated**	No data	No data	1 (2.2)	1.1 (2.6)	4.3 (9)	5.7 (12)	0.4 (5.7)
**Unresolved**	No data	No data	2.4 (5.6)	0.1 (0.3)	2 (4.3)	0.2 (0.4)	0 (0.6)
**Not Known**	No data	No data	0 (0)	0 (0)	4.8 (10)	4.7 (9.9)	0.5 (7.6)
**Data not recorded**	No data	No data	No data	No data	No data	No data	0.1 (1.3)
**Value outside of national code**	No data	No data	No data	No data	No data	No data	0 (0.1)

Includes all ethnic categories available within the data sources (e.g., Not Stated, Not Known) or which have been derived (i.e., Unresolved).

All counts for ECIA, GDPPR, HES, and TT are based on the data sets linked to Census 2021. Census 2021 counts are based on unlinked data set. For GDPPR, HES, and TT data sources, these data refer to when no reallocation was applied. No data denotes the category did not exist within the data source, rather than a count of 0.

Counts are presented in millions and rounded to the nearest hundredth thousand. Percentage data rounded to 1dp and overall percentage may not sum to exactly 100.

Data are sorted in count descending order for Census 2021.

ECIA, Ethnic Category Information Asset; GDPPR, GPES Data for Pandemic Planning and Research; HES, Hospital Episode Statistics; TT, Talking Therapies.

### Overall comparison

Overall agreement ranged from 86.4% for HES-recency to 92.4% for TT for the 18-category ethnic groups (**S4 Table**). Similar patterns were seen with the 5-category ethnic groups but agreement was higher for all sources (ranging from 93.3% for HES-recency to 96.4% for TT).

### Individual ethnic category comparison

**Fig 2:**
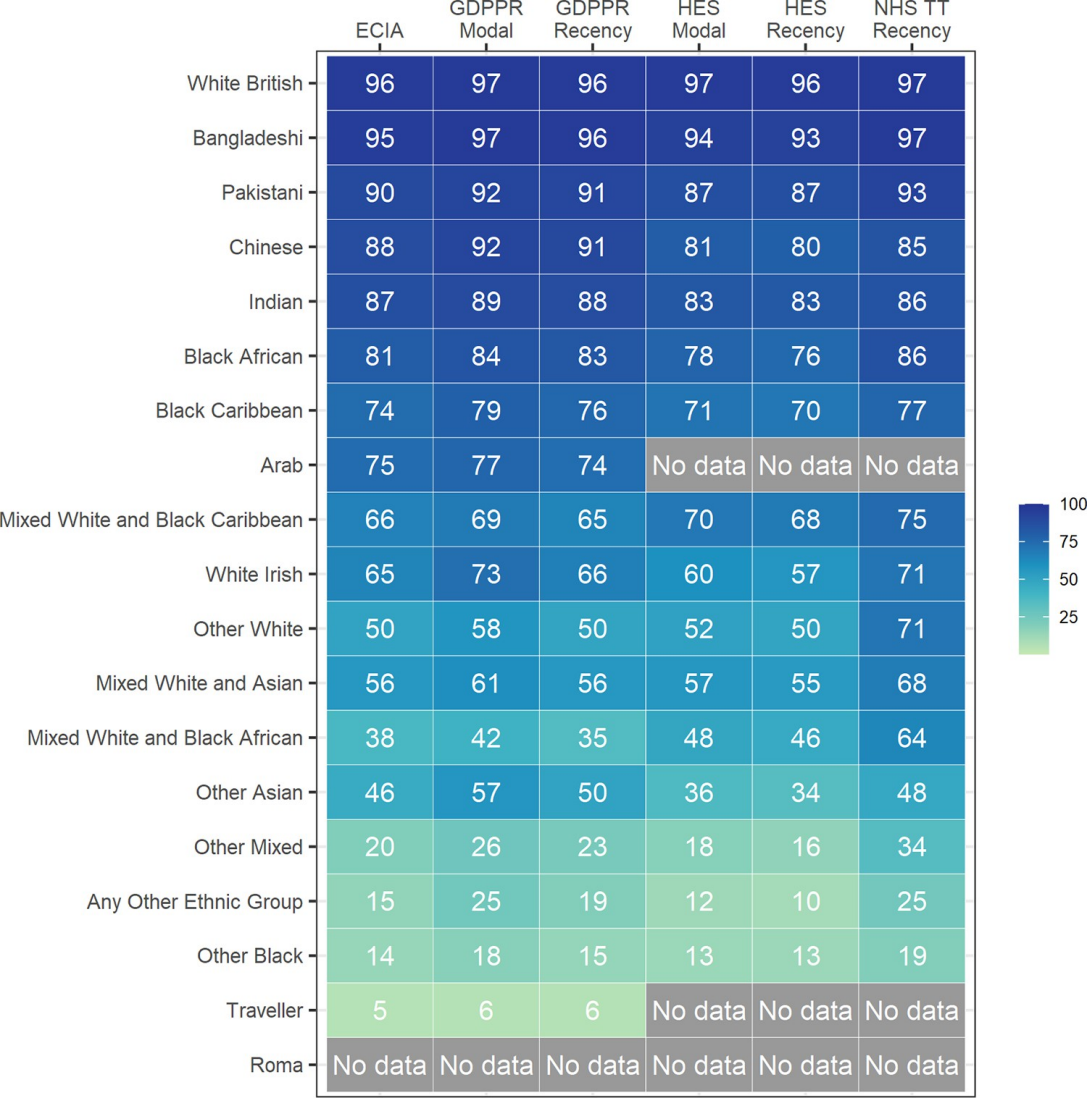
Percentage of agreement between health datasets and Census 2021 using 18-category ethnicities, England. Data presented is percentage (%). Agreement is based on linked individuals with a stated ethnicity in the relevant health dataset and Census 2021. “Not Stated,” “Not Known,” or “Unresolved” categories were excluded from the agreement calculation. The population included is therefore different for each data source. For each source, the health data ethnic group totals have been used as denominators when calculating percentages. The Arab and Traveller ethnic group categories are not available in HES or NHS TT, so agreement for these categories are only presented for ECIA and GDPPR. The Roma ethnic group is not available for any data set. For GDPPR, HES, and TT data sources, these data refer to when the Unknown only reallocation methodology has been applied. ECIA, Ethnic Category Information Asset; GDPPR, GPES Data for Pandemic Planning and Research; HES, Hospital Episode Statistics; NHS TT, National Health Service Talking Therapies.

**Fig 2** shows the agreement between each NHSE data source with Census 2021 for the 18-category ethnic classifications. Across all data sources, the White British category consistently showed the highest level of agreement (≥96%). The Bangladeshi category showed the second highest levels of agreement across all sources (≥93%), with the same level of agreement as the White British category for GDPPR and TT sources. Pakistani (≥87%), Indian (≥83%), and Chinese (≥80%) categories showed the next highest levels of agreement across all data sources. Black African and Black Caribbean categories showed agreement with Census 2021 ranging from 70% to 86% across all data sources. The ethnic category with the lowest agreement across the ECIA and GDPPR data sets was the Gypsy or Irish Traveller category (≤6%). The Gypsy or Irish Traveller ethnic group was not available within HES or TT, which use 16 ethnic categories for reporting (**S2 Table**). The ethnic categories with the lowest level of agreement within HES and TT data sources were the Any Other Ethnic Group (10% to 25%) and Other Black (13% to 19%) categories, respectively. Agreement was generally lower for all Mixed (≤75%) and Other (≤71%) ethnic categories across all data sources.

While patterns of agreement with census were similar across all health data sources, GDPPR modal and TT generally reported the highest levels of agreement for most ethnic categories. This was particularly pronounced for Mixed and Other ethnic categories, with the TT data source generally reporting the highest levels of agreement for these ethnic categories. When comparing GDPPR with HES, GDPPR reported higher agreement with census for all ethnic categories, except for Mixed: White and Black African and Mixed: White and Black Caribbean.

Patterns of agreement were similar when aggregating ethnicity to 5-category ethnic groupings (S**1 Fig**). For White, Asian, and Black categories, the 5-category ethnic groupings showed agreement ≥88% across all sources, meaning differences in ethnicity recording are predominantly within the same 5-category grouping (e.g., Black African, Black Caribbean, and Other Black). Mixed and Other category agreement was mostly higher compared with the 18-category results of the same disaggregated categories, but still showed lower agreement overall, meaning the differences in ethnicity recording are less likely to be within the same 5-category ethnic groupings (e.g. Other Asian and Any Other Ethnic Group).

Crosstabulations displaying the count and percentage agreement for 5- and 18-category ethnic groups between each data source and Census 2021 can be found in the Supporting information (**S5–S16 Tables**). Results from our sensitivity analyses mirror our main results (**S2–S3 Figs and S17–S18 Tables**). Sensitivity and PPV for each data source are shown in **S19 Table**.

### Reallocation of ethnicity in episodic data (GDPPR, HES, and TT)

**Fig 3:**
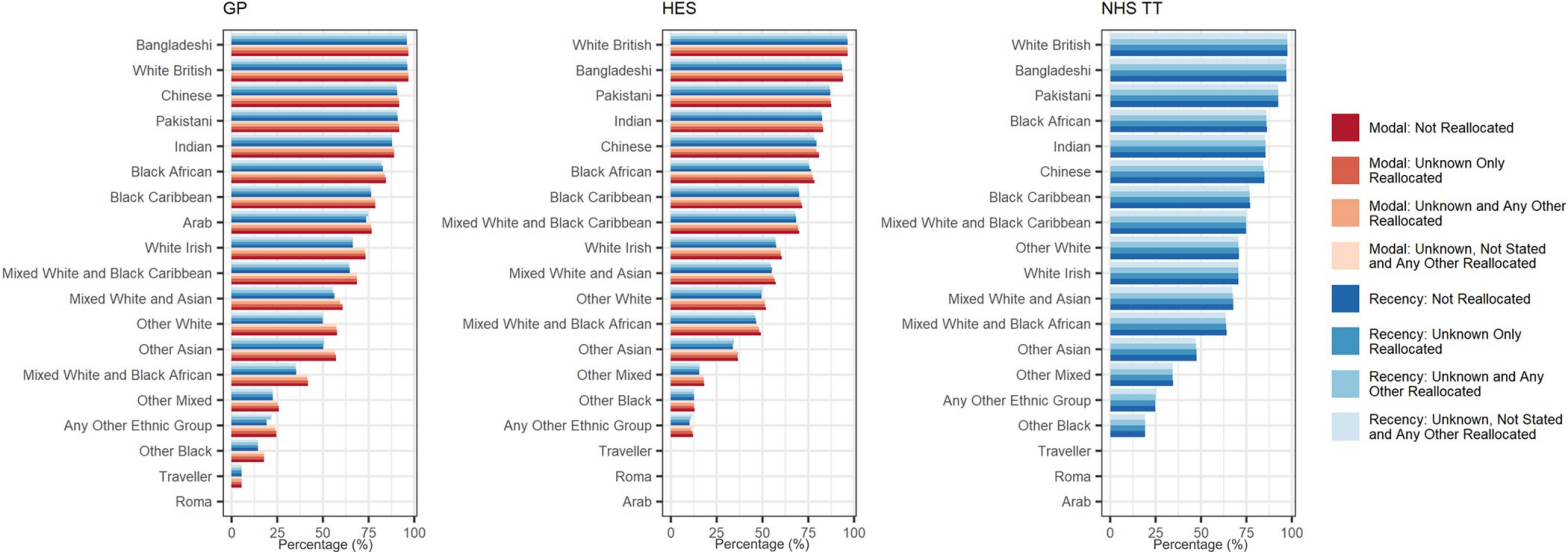
Impact of reallocation methodologies on percentage agreement with Census 2021 for both recency and modal definitions in GDPPR, HES, and TT, by 18-category ethnic groups, England. Data presented is percentage (%). Red denotes modal definitions; blue denotes recency definitions. Lighter shade colour denotes more reallocation of ethnic categories (as indexed in key). As described in the methods section in further detail, it was not possible to apply the modal definition to the NHS TT data source. Agreement is based on linked individuals with a stated ethnicity in the relevant reallocation methodology GDPPR data set and Census 2021. The population included is therefore different for each data source. For each reallocation methodology, the health data ethnic group totals have been used as denominators when calculating percentages. The Roma ethnic group category is not available in GDPPR. The Arab, Traveller, and Roma ethnic group categories are not available in HES and TT. Data are presented in ascending agreement order for each data source. Therefore, the order of ethnic categories along the y axis may differ for each data source. GDPPR, GPES Data for Pandemic Planning and Research; HES, Hospital Episode Statistics; NHS TT, National Health Service Talking Therapies.

**Fig 3** shows the impact of using different reallocation methods to assign an ethnicity to an individual in GDPPR, HES, and TT, respectively. For all data sources, no notable changes in agreement with Census 2021 were seen between any of the reallocation methodologies applied for either modal or recency definitions, with agreement per ethnic group being similar for all levels of reallocation. Some differences in coverage were seen between the reallocation methodologies (**S20 Table**). The modal definition could not be derived for TT.

### Recency vs. modal

In GDPPR, the recency method to assign an ethnic category provides greater coverage compared with modal method (**S20 Table**). In HES, recency and modal provided varied coverage dependent upon the reallocation methodology applied. When investigating accuracy, irrespective of coverage (i.e., the reallocation method used), modal definitions reported higher agreement with Census 2021 than recency. The differences between modal and recency agreement was pronounced in White Irish, all Mixed and all Other ethnic categories (**Fig 3**).

## Discussion

Using Census 2021, GDPPR, HES, ECIA, and TT data sources, we compared the agreement in ethnicity recording between the health administrative data sources and census at individual record level. Across all comparisons with self-reported, Census 2021 ethnicity, we found that the level of concordance in the 18-category ethnicity recording is highly variable between ethnic groups, with particularly high levels of discordance found for White: Gypsy or Irish Traveller (not available in HES or TT), White Irish, all Mixed (Other Mixed; White and Black African; White and Asian; White and Black Caribbean) and all Other (Any Other Ethnic Group; Other Black; Other Mixed; Other Asian; Other White) ethnic categories. The highest levels of agreement across all data sources were found in the White British ethnic category, followed by South Asian (Bangladeshi, Pakistani, and Indian) and Chinese ethnic categories. We also found that, while patterns of agreement were generally found to be similar across all data sources, for most ethnic groups, agreement was lower in HES and higher in GDPPR and TT.

### In context of the literature

Our results are similar to another population-level analysis that investigated agreement between the 5-category ethnic groups in 2011 Census and HES 2009–2011 [9]: the results from this analysis evidenced that the White ethnic group had the highest level of agreement between the sources (98.7%), with levels of agreement in Asian and Black ethnic groups at 87.9% and 84.7%, respectively. The lowest levels of agreement were found in the Mixed (36.9%) and Other (27.5%) ethnic groups [9]. While our study reports the same pattern of agreement for Census 2021 and HES (recency and modal) in the 5-category ethnic categories, we report different levels of agreement which were lower for the Other ethnic category and higher for the Black and Mixed categories. This may be due to the different time period used in our analysis for HES data and our updated census data. For example, it was found that Mixed and Other ethnic groups were more likely to change their ethnic category over time than White, Black, and Asian ethnic groups between 2001 and 2011 [28]. Therefore, similar patterns may have followed between 2011 and 2021, which may partially explain some differences seen in our results. Further, there have been drives to improve ethnicity coding in HES since 2011 [7], which may further explain this difference. Our results further align with previous research that investigated the accuracy of ethnicity coding between HES and the English Cancer Patient Experience survey [27]. Results from this study highlighted that the probability of concordant classication with the census data was highest in White ethnic groups, followed by high levels of agreement in Bangladeshi, Indian, Pakistani, Chinese, Black African, and Black Caribbean individuals [27]. Mixed and Other ethnic groups reported similarly low agreement in the aforementioned study compared with our own. In addition, our findings align with a similar population-level analysis which examined the quality of ethnicity coding within Scottish health records, with misclassification of ethnic categories being higher in all ethnic minority groups compared with the White Scottish category, particularly Other and Gypsy or Irish Traveller categories [8]. Our results also broadly align with previous studies from US data which [29,30], while not directly comparable due to the differing healthcare systems and how ethnicity may be interpreted between countries, found that levels of agreement were higher in White individuals compared with individuals from a minority ethnic group [29,30]. The higher misclassification of ethnic category in ethnic minority groups is likely down to a multitude of reasons and is not well understood. A suggested reason for higher misclassification within certain ethnic groups is the lack of standardisation of data collection processes across multiple healthcare settings and systems [2,31].

Our study also found that levels of agreement with Census 2021 were generally similar across all data sources for each ethnic category. However, HES did report lower levels of agreement than ECIA and particularly GDPPR and TT across most ethnic categories. This may be explained by a previous study which highlighted that HES outpatients and A&E data sets have poor consistency of ethnicity coding [11,12]. Furthermore, our data showed that HES has high levels of coding for Not Known and Not Stated categories compared with other health administrative data sources. However, even in analysis where we reallocated Not Known and Not Stated categories, HES still had lower agreement with census than other data sources. Interestingly, while the ECIA is an amalgamation of HES and GDPPR ethnicity data to improve coverage for the entire English population [18], our findings report that the agreement with census is similar or worse compared with using GDPPR individually. This may be expected because GDPPR data has higher agreement than HES and therefore, using HES data to fill in gaps in GDPPR data would result in lower agreement (though having higher coverage). This is an important finding given that the ECIA is the most complete source of ethnicity information covering the whole population available to NHSE for ethnicity analyses.

### Strengths and limitations

This study took advantage of a unique opportunity to explore linked Census 2021 data with multiple routinely collected NHSE data sources. This was the first time ethnicity data from GP and ECIA sources have been linked to census at person-level to assess the accuracy of ethnicity recording in these data sources. Further, a high proportion of census records were able to be linked to an NHS number (95.75%), which allowed for a large number of individuals to be included in the analysis and provide a representative sample of the population of England. The novelty of this analysis was being able to use Census 2021 as a gold standard for ethnicity data because of its self-reported nature. Although self-reported ethnicity may be prone to certain biases [32,33], it is generally considered one of the most robust methods to collect ethnicity information [15]. Self-reported ethnicity may change with time and age [28,34]. However, the impact of this on our analysis is limited because of Census 2021 data being the most up to date ethnicity data available for the entire population of England at the time of analysis. Further, we were able to identify individuals with imputed census ethnicity and remove them from the analysis.

This study does have some limitations, however. An important limitation of this work is, because all individuals within our analysis had to have a stated ethnicity recorded in the Census 2021, it excluded people who did not take part in the census (estimated to be 3% of the population), recent migrants, and people who could not be linked to the NHS PDS, which may affect representativeness of the population used. However, our data set included 90.8% of the population living in England on Census Day. In addition, a limitation of the linkage approach used is that linkage rates vary between ethnic groups [20,35]. However, this methodology does result in a linked population with a high coverage of England that is implemented in many other ONS publications. Linkage between sources may also sometimes be imperfect and result in false positive linkage. Further, the demographic characteristics of the populations across the linked data sources may vary, which could explain some of the observed differences in agreement. Further, it is noted that some ethnicity responses in the census data may be provided by a proxy, for example, a parent on behalf of a child who cannot respond for themselves. Proxy reporting does not only affect census data as the health data sources are likely to also contain some proxy responses affecting the comparisons. In addition, it has been reported that ethnic category is sometimes recorded by NHS staff without asking the patient or there is a reluctance from staff to ask about ethnicity within healthcare environments [11]. The context in which ethnicity information was collected is not available within EHRs and therefore, identifying whether a record is truly self-reported or completed by healthcare staff is not possible to assess. Therefore, understanding potential differences in how people self-identify versus how they present to others is not possible in the available data.

### Implications for policymakers and researchers

There is discrepancy in how major national (quasi)regulatory agencies handle ethnicity data, for example, NHSE currently use the most recent ethnicity an individual has recorded to determine their ethnicity [24], while the Office for Health Improvement and Disparities (OHID) use modal [36]. Our study illustrates that the impact of applying different reallocation methodologies to assign an ethnic category had little impact on agreement with census per ethnic group.

However, applying reallocation methodologies versus not applying them increased the coverage of people assigned a stated ethnic category (i.e., not a recording of Not Known or Not Stated). This increase in coverage was particularly high in HES, with the most reallocated iteration increasing the number of people with a stated ethnic category by 6.5m (recency) and 6.7m (modal), respectively. Previous work has investigated the impact of reallocating ethnic categories to account for Not Known recordings and an over-coding of Any Other Ethnic Group [37] and found that applying reallocation methodologies will increase coverage but marginally reduce accuracy for some ethnic categories.

As such, the results presented here, in combination with previous research, confirm that using the most recent or most frequent approach to assigning ethnic categories both provide suitable options for deriving ethnic group data.

In a broader context, if some individuals’ ethnicity data is not being recorded accurately within health administrative data sources, it may have implications for healthcare planning and resource allocation. If individuals’ ethnicity data is being misclassified on a population scale, it may lead to under- or overestimation of health outcomes or conditions in certain ethnic groups, and potentially misrepresent the true pattern and quantity across ethnic groups. Previous research has found that the misclassification of ethnicity within Scottish records concealed the high-risk of severe COVID-19 among the Gypsy and Irish Traveller ethnic group, and the under- and overestimation of risk in other ethnic groups [8]. This misclassification may have knock on effects in healthcare planning, public health and healthcare resource allocation, and health policy formation, as well as the monitoring of ethnic health inequalities. It may lead to the continuation or exacerbation of ethnic health inequalities in certain ethnic groups.

Furthermore, our findings provide ground for other analysts to replicate our methods for wider use and further develop improvements. While this analysis is based on English data, the understanding and methodologies can be applied to other data sources and data from across the UK. Greater understanding of ethnicity coding and its limitations may lead to developing methods and consensus for analysts on how to best use ethnicity data for health and administrative analysis and statistics, ultimately leading to improvements in analyses and statistics which inform policies that aim to reduce ethnic health inequalities in England. This is particularly timely given healthcare providers and the UK Government commitment to reducing ethnic health disparities [38,39]. It is acknowledged that improving the quality of ethnicity data in administrative health data sources is a multifaceted problem. It likely requires a coordinated approach from many different organisations, and includes targeting a standardised definition on the term ethnicity, and standardising the methods that ethnicity data is collected, recorded, and processed. The standardisation of these factors would likely improve people’s understanding and the quality of ethnicity data.

## Conclusions

This population-level study of residents in England demonstrates that certain ethnic categories across multiple health administrative data sources have high discordance with Census 2021 ethnic categories. Agreement in groups of individuals with Mixed and Other ethnic categories were consistently found to be lower across all health data sources, with individuals who were classified as Gypsy or Irish Traveller also reporting particularly low levels of agreement with census. However, it must be acknowledged these health administrative data sources have been used to good effect to date, ethnicity coding quality issues notwithstanding. Not least evidenced than during the pandemic. They should be continued to be utilised to investigate ethnic inequalities in health and access, while simultaneously improving the data quality. This study demonstrates the value of data linkage by reporting the completeness and accuracy of ethnicity recordings across data sources. Future studies should determine the difference in health outcomes in those with discordant ethnicity recording between different data sets, the sociodemographic characteristics of those who have discordant (with census) or missing ethnicity data, and how ethnicity data may change longitudinally.

### Ethics approval and consent to participate

Ethical approval was obtained from the National Statistician’s Data Ethics Advisory Committee (NSDEC(20)12). This study involved secondary use of health administrative data sets. Therefore, informed consent was not required.

## Supporting information

S1 STROBE Checklist Checklist of items that should be included in reports of observational studies. (DOCX)

S1 Table Count of people in the linked datasets created to compare the quality of ethnicity recording in health data sources with that in Census 2021, England. (DOCX)

S2 Table Description of ethnic categories from Census 2011, GDPPR, HES, ECIA, and TT health admin data sources. (DOCX)

S3 Table Census 2021 categories for England aligned to GSS ethnic harmonised standard. (DOCX)

S4 Table Overall agreement by health data source in comparison with Census 2021, using 18-category and 5-category ethnic categories, England. (DOCX)

S5 Table Crosstabulations (A) and level of agreement (B) for 18-category ethnicity coding in individuals in the linked Census 2021-ECIA data set. (DOCX)

S6 Table Crosstabulations (A) and levels of agreement (B) for 18-category ethnicity coding in individuals in the linked Census 2021-GDPPR recency unknown only data set. (DOCX)

S7 Table Crosstabulations (A) and level of agreement (B) for 18-category ethnicity coding in individuals in the linked Census 2021-GDPPR modal unknown only data set. (DOCX)

S8 Table Crosstabulations (A) and level of agreement (B) for 18-category ethnicity coding in individuals in the linked Census 2021-HES recency unknown only data set. (DOCX)

S9 Table Crosstabulations (A) and level of agreement (B) for 18-category ethnicity coding in individuals in the linked Census 2021-HES modal unknown only data set. (DOCX)

S10 Table Crosstabulations (A) and level of agreement (B) for 18-category ethnicity coding in individuals in the linked Census 2021-TT recency unknown only data set. (DOCX)

S11 Table Crosstabulations (A) and level of agreement (B) for 5-category ethnicity coding in individuals in the linked Census 2021-ECIA data set. (DOCX)

S12 Table Crosstabulations (A) and level of agreement (B) for 5-category ethnicity coding in individuals in the linked Census 2021-GDPPR recency unknown only data set. (DOCX)

S13 Table Crosstabulations (A) and level of agreement (B) for 5-category ethnicity coding in individuals in the linked Census 2021-GDPPR modal unknown only dataset. (DOCX)

S14 Table Crosstabulations (A) and level of agreement (B) for 5-category ethnicity coding in individuals in the linked Census 2021-HES recency unknown only data set. (DOCX)

S15 Table Crosstabulations (A) and level of agreement (B) for 5-category ethnicity coding in individuals in the linked Census 2021-HES modal unknown only data set. (DOCX)

S16 Table Crosstabulations (A) and level of agreement (B) for 5-category ethnicity coding in individuals in the linked Census 2021-TT recency unknown only data set. (DOCX)

S17 Table Percentage of agreement between health datasets and Census 2021 using 18-category ethnicities in a sensitivity analysis restricting the population to people with a stated ethnic category in each of Census 2021, GDPPR, and HES data sources only, England. (DOCX)

S18 Table Percentage of agreement between health data sets and Census 2021 using 5-category ethnicities in a sensitivity analysis restricting the population to people with a stated ethnic category in each of Census 2021, GDPPR, and HES data sources only, England. (DOCX)

S19 Table Comparison of sensitivity and positive predictive value in 18-category ethnicity categories within Census 2021 to the ECIA, GDPPR, HES, and TT data sources, England. (DOCX)

S20 Table Count of people in the linked data sets created to compare consistency of ethnicity recording in health sources with the Census 2021, England. (DOCX)

S1 Fig Percentage of agreement between health data sets and Census 2021 using 5-category ethnicities, England. (DOCX)

S2 Fig Percentage of agreement between health datasets and Census 2021 using 18-category ethnicities in a sensitivity analysis restricting the back series of data to 1 April 2015, England. (DOCX)

S3 Fig Percentage of agreement between health data sets and Census 2021 using 5-category ethnicities in a sensitivity analysis restricting the back series of data to 1 April 2015, England. (DOCX)

## Abbreviations

APC: Admitted Patient Care
CPRD: Clinical Practice Research Datalink
ECDS: Emergency Care Dataset
ECIA: Ethnic Category Information Asset
EHR: electronic health record
GDPPR: GPES Data for Pandemic Planning and Research
GPES: General Practice Extraction Service
HES: Hospital Episode Statistics
IAPT: Improving Access to Psychological Therapies
NHS: National Health Service
OHID: Office for Health Improvement and Disparities
PDS: Personal Demographics Service
PPV: positive predictive value
TT: Talking Therapies
