# Supplementary material for: Understanding the quality of ethnicity data recorded in health-related administrative data sources compared with Census 2021 in England
Source: PLoS Med. 2025 Feb 26;22(2):e1004507. doi: 10.1371/journal.pmed.1004507 (PMC11864522; doi:10.1371/journal.pmed.1004507)
Supplement: S1 STROBE Checklist — (DOCX) [file pmed.1004507.s001.docx]

STROBE Statement—checklist of items that should be included in reports of observational studies

|  | Item No. | Recommendation | Page  No. | Relevant text from manuscript |
| --- | --- | --- | --- | --- |
| **Title and abstract** | 1 | (*a*) Indicate the study’s design with a commonly used term in the title or the abstract | 1 | Title section |
|  |  | (*b*) Provide in the abstract an informative and balanced summary of what was done and what was found | 3 | Abstract section |
| Introduction | | | |  |
| Background/rationale | 2 | Explain the scientific background and rationale for the investigation being reported | 5 | Paras 1 and 2 of intro section |
| Objectives | 3 | State specific objectives, including any prespecified hypotheses | 5 | Para 3 of intro section |
| Methods | | | |  |
| Study design | 4 | Present key elements of study design early in the paper | 7-9 | Data sources and study pop, data linkage, ethnicity definitions with each data source sections in methods |
| Setting | 5 | Describe the setting, locations, and relevant dates, including periods of recruitment, exposure, follow-up, and data collection | 7-9 | Data sources and study pop, data linkage, ethnicity definitions with each data source sections in methods |
| Participants | 6 | (*a*) *Cohort study*—Give the eligibility criteria, and the sources and methods of selection of participants. Describe methods of follow-up  *Case-control study*—Give the eligibility criteria, and the sources and methods of case ascertainment and control selection. Give the rationale for the choice of cases and controls  *Cross-sectional study*—Give the eligibility criteria, and the sources and methods of selection of participants | 7-10 | Fully detailed throughout entire methods section. Eligibility criteria and follow-up period of data is in Data Linkage section. Definition of ethnicity (main exposure) is in Ethnicity definitions section |
|  |  | (*b*) *Cohort study*—For matched studies, give matching criteria and number of exposed and unexposed  *Case-control study*—For matched studies, give matching criteria and the number of controls per case | N/A |  |
| Variables | 7 | Clearly define all outcomes, exposures, predictors, potential confounders, and effect modifiers. Give diagnostic criteria, if applicable | 7-10 | Exposures and definition/manipulation of definitions detailed in Ethnicity definitions, Handling multiple ethnicity records per person and reallocating ethnicity records section. Analysis detailed in Statistical Analysis section |
| Data sources/ measurement | 8* | For each variable of interest, give sources of data and details of methods of assessment (measurement). Describe comparability of assessment methods if there is more than one group | 7-10 | Details on data sources and methods of assignment detailed in Data sources and study population, data linkage sections. Comparability of assessment methods described in handling multiple ethnicity records per person and reallocating ethnicity records. Statistical analysis section further details comparison of methods |
| Bias | 9 | Describe any efforts to address potential sources of bias | 10 | Detailed in Statistical analysis section |
| Study size | 10 | Explain how the study size was arrived at | 7-8, 25 | Detailed in Data linkage section and in Figure 1 |

Continued on next page

| Quantitative variables | 11 | Explain how quantitative variables were handled in the analyses. If applicable, describe which groupings were chosen and why | 8-10 | Detailed in Handling multiple ethnicity records per person, reallocating ethnicity records and statistical analysis sections |
| --- | --- | --- | --- | --- |
| Statistical methods | 12 | (*a*) Describe all statistical methods, including those used to control for confounding | 10 | Stats analysis section |
|  |  | (*b*) Describe any methods used to examine subgroups and interactions | 10 | Stats analysis section |
|  |  | (*c*) Explain how missing data were addressed | 7-9 | Detailed in Data Linkage section. Also in Handling multiple ethnicity records per person and reallocating ethnicity records sections |
|  |  | (*d*) *Cohort study*—If applicable, explain how loss to follow-up was addressed  *Case-control study*—If applicable, explain how matching of cases and controls was addressed  *Cross-sectional study*—If applicable, describe analytical methods taking account of sampling strategy | N/A |  |
|  |  | (*e*) Describe any sensitivity analyses | 10 | Stats analysis section |
| Results | | | | |
| Participants | 13* | (a) Report numbers of individuals at each stage of study—eg numbers potentially eligible, examined for eligibility, confirmed eligible, included in the study, completing follow-up, and analysed | 25 | Detailed in Figure 1 |
|  |  | (b) Give reasons for non-participation at each stage | 7-8 | Data Linkage subsection |
|  |  | (c) Consider use of a flow diagram | 25 | Included – Figure 1 |
| Descriptive data | 14* | (a) Give characteristics of study participants (eg demographic, clinical, social) and information on exposures and potential confounders | 11, 23 | Details of main exposure (ethnicity) only as we are comparing the quality of ethnicity in the data sources, rather than focusing on health outcomes 1^st^ para of results. Also in table 1. |
|  |  | (b) Indicate number of participants with missing data for each variable of interest | 25 | This can be calculated from flowchart in Figure 1 |
|  |  | (c) *Cohort study*—Summarise follow-up time (eg, average and total amount) | N/A |  |
| Outcome data | 15* | *Cohort study*—Report numbers of outcome events or summary measures over time | N/A |  |
|  |  | *Case-control study—*Report numbers in each exposure category, or summary measures of exposure | N/A |  |
|  |  | *Cross-sectional study—*Report numbers of outcome events or summary measures | N/A |  |
| Main results | 16 | (*a*) Give unadjusted estimates and, if applicable, confounder-adjusted estimates and their precision (eg, 95% confidence interval). Make clear which confounders were adjusted for and why they were included | 11-12, 26-27 | In para 2, 3, 4, 5, 6 and 7 of results. In Figures 2 and 3 too |
|  |  | (*b*) Report category boundaries when continuous variables were categorized | N/A |  |
|  |  | (*c*) If relevant, consider translating estimates of relative risk into absolute risk for a meaningful time period | N/A |  |

Continued on next page

| Other analyses | 17 | Report other analyses done—eg analyses of subgroups and interactions, and sensitivity analyses | 10 | Para 4 of statistical analysis section |
| --- | --- | --- | --- | --- |
| Discussion | | | | |
| Key results | 18 | Summarise key results with reference to study objectives | 12 | Para 1 of discussion section |
| Limitations | 19 | Discuss limitations of the study, taking into account sources of potential bias or imprecision. Discuss both direction and magnitude of any potential bias | 14-15 | Within Strengths and Limitations section of Discussion. |
| Interpretation | 20 | Give a cautious overall interpretation of results considering objectives, limitations, multiplicity of analyses, results from similar studies, and other relevant evidence | 13-14 | Included in the In Context of the Literature and Strength and Limitations, and Implications sections of Discussion |
| Generalisability | 21 | Discuss the generalisability (external validity) of the study results | 14-16 | Included in Strength and Limitations, and Implications sections of Discussion, and the Conclusion |
| Other information | |  | | |
| Funding | 22 | Give the source of funding and the role of the funders for the present study and, if applicable, for the original study on which the present article is based | 17 | In funding section |

*Give information separately for cases and controls in case-control studies and, if applicable, for exposed and unexposed groups in cohort and cross-sectional studies.

**Note:** An Explanation and Elaboration article discusses each checklist item and gives methodological background and published examples of transparent reporting. The STROBE checklist is best used in conjunction with this article (freely available on the Web sites of PLoS Medicine at http://www.plosmedicine.org/, Annals of Internal Medicine at http://www.annals.org/, and Epidemiology at http://www.epidem.com/). Information on the STROBE Initiative is available at www.strobe-statement.org.
