# Supplementary material for: Understanding the quality of ethnicity data recorded in health-related administrative data sources compared with Census 2021 in England
Source: PLoS Med. 2025 Feb 26;22(2):e1004507. doi: 10.1371/journal.pmed.1004507 (PMC11864522; doi:10.1371/journal.pmed.1004507)
Supplement: S1 Table — (DOCX) [file pmed.1004507.s002.docx]

# **Table S1.** Count of people in the linked datasets created to compare the quality of ethnicity recording in health data sources with that in Census 2021, England.

| **Linked dataset, ethnicity allocation method** | **Count of people in each linked dataset** | | **Count of people in linked dataset with a stated ethnicity in both health and census sources** | |
| --- | --- | --- | --- | --- |
|  | ***Millions (n)*** | ***Percentage of the population of England on Census day 2021 (%)*** | ***Millions (n)*** | ***Percentage of the population of England on Census day 2021 (%)*** |
| **Linked census-ECIA** | 47.4 | 83.9 | 47.4 | 83.9 |
| **Linked census-GDPPR, modal** | 43.5 | 77.0 | 40.1 | 71.0 |
| **Linked census-GDPPR, recency** | 43.5 | 77.0 | 42.2 | 74.7 |
| **Linked census-HES, modal** | 47.8 | 84.6 | 40.1 | 71.0 |
| **Linked census-HES, recency** | 47.8 | 84.6 | 39.7 | 70.3 |
| **Linked census-TT, recency** | 6.3 | 11.2 | 5.4 | 9.6 |

For GDPPR, HES and TT data sources, these data refer to when the Unknown only reallocation methodology has been applied.
No modal definition was derived for NHS Talking Therapies (TT).
A stated ethnicity in both sources excludes individuals whose ethnicity from the health data was “Not Known”, “Not Stated” or “Unresolved” after applying the recency or modal methodologies.
