# Supplementary material for: Understanding the quality of ethnicity data recorded in health-related administrative data sources compared with Census 2021 in England
Source: PLoS Med. 2025 Feb 26;22(2):e1004507. doi: 10.1371/journal.pmed.1004507 (PMC11864522; doi:10.1371/journal.pmed.1004507)
Supplement: S2 Table — (DOCX) [file pmed.1004507.s003.docx]

# **Table S2**. Description of ethnic categories from Census 2011, GDPPR, HES, ECIA and TT health admin data sources.

| **Census 2021** | **GDPPR** | **HES** | **ECIA** | **TT** |
| --- | --- | --- | --- | --- |
| White: English/Welsh/Scottish/Northern Irish/British | British | British (White) | White: British | White: British |
| White: Irish | Irish | Irish (White) | White: Irish | White: Irish |
| White: Gypsy or Irish Traveller | Traveller |  | Other: Traveller |  |
| White: Roma |  |  |  |  |
| White: Other White | Any other White background | Any other White background | White: Any other White background | White: Any other White background |
| Mixed/multiple ethnic groups: White and Black Caribbean | White and Black Caribbean | White and Black Caribbean (Mixed) | Mixed: White and Black Caribbean | Mixed: White and Black Caribbean |
| Mixed/multiple ethnic groups: White and Black African | White and Black African | White and Black African (Mixed) | Mixed: White and Black African | Mixed: White and Black African |
| Mixed/multiple ethnic groups: White and Asian | White and Asian | White and Asian (Mixed) | Mixed: White and Asian | Mixed: White and Asian |
| Mixed/multiple ethnic groups: Other Mixed | Any other Mixed background | Any other Mixed background | Mixed: Any other mixed background | Mixed: Any other mixed background |
| Asian/Asian British: Indian | Indian | Indian (Asian or Asian British) | Asian: Indian | Asian: Indian |
| Asian/Asian British: Pakistani | Pakistani | Pakistani (Asian or Asian British) | Asian: Pakistani | Asian: Pakistani |
| Asian/Asian British: Bangladeshi | Bangladeshi | Bangladeshi (Asian or Asian British) | Asian: Bangladeshi | Asian: Bangladeshi |
| Asian/Asian British: Chinese | Chinese | Chinese (Other ethnic group) | Other: Chinese | Other: Chinese |
| Asian/Asian British: Other Asian | Any other Asian background | Any other Asian background | Asian: Any other Asian background | Asian: Any other Asian background |
| Black/African/Caribbean/Black British: African | African | African (Black or Black British) | Black: African | Black: African |
| Black/African/Caribbean/Black British: Caribbean | Caribbean | Caribbean (Black or Black British) | Black: Caribbean | Black: Caribbean |
| Black/African/Caribbean/Black British: Other Black | Any other Black background | Any other Black background | Black: Any other Black background | Black: Any other Black background |
| Other ethnic group: Arab | Arab |  | Other: Arab |  |
| Other ethnic group: Any other ethnic group | Any other ethnic group | Any other ethnic group | Other: Any other ethnic group | Other: Any other ethnic group |
