# Supplementary material for: Understanding the quality of ethnicity data recorded in health-related administrative data sources compared with Census 2021 in England
Source: PLoS Med. 2025 Feb 26;22(2):e1004507. doi: 10.1371/journal.pmed.1004507 (PMC11864522; doi:10.1371/journal.pmed.1004507)
Supplement: S3 Table — (DOCX) [file pmed.1004507.s004.docx]

# **Table S3**. Census 2021 categories for England aligned to GSS ethnic harmonised standard.

| **GSS harmonised standard** | **Census 2021** |
| --- | --- |
| White | White: English/Welsh/Scottish/Northern Irish/British |
|  | White: Irish |
|  | White: Gypsy or Irish Traveller |
|  | White: Roma |
|  | White: Other White |
| Mixed / Multiple ethnic groups | Mixed/multiple ethnic groups: White and Black Caribbean |
|  | Mixed/multiple ethnic groups: White and Black African |
|  | Mixed/multiple ethnic groups: White and Asian |
|  | Mixed/multiple ethnic groups: Other Mixed |
| Asian / Asian British | Asian/Asian British: Indian |
|  | Asian/Asian British: Pakistani |
|  | Asian/Asian British: Bangladeshi |
|  | Asian/Asian British: Chinese |
|  | Asian/Asian British: Other Asian |
| Black / African / Caribbean / Black British | Black/African/Caribbean/Black British: African |
|  | Black/African/Caribbean/Black British: Caribbean |
|  | Black/African/Caribbean/Black British: Other Black |
| Other ethnic group | Other ethnic group: Arab |
|  | Other ethnic group: Any other ethnic group |

ű
