# Supplementary material for: Understanding the quality of ethnicity data recorded in health-related administrative data sources compared with Census 2021 in England
Source: PLoS Med. 2025 Feb 26;22(2):e1004507. doi: 10.1371/journal.pmed.1004507 (PMC11864522; doi:10.1371/journal.pmed.1004507)
Supplement: S4 Table — (DOCX) [file pmed.1004507.s005.docx]

# **Table S4.** Overall agreement by health data source in comparison with Census 2021, using 18-category and 5-category ethnic categories, England.

| **Dataset, ethnicity allocation method** | **Overall agreement for 18-category ethnic groups (%)** | **Overall agreement for 5-category ethnic groups (%)** |
| --- | --- | --- |
| **ECIA** | 86.7 | 94.0 |
| **GDPPR, modal** | 89.9 | 95.7 |
| **GDPPR, recency** | 87.0 | 94.6 |
| **HES, modal** | 87.6 | 94.1 |
| **HES, recency** | 86.4 | 93.3 |
| **TT, recency** | 92.4 | 96.4 |

Data are presented as percentage (%).
For GDPPR, HES and TT data sources, these data refer to when the Unknown only reallocation methodology has been applied.
The percentages are based on individuals with a stated ethnicity in both the health data source and Census 2021; those whose ethnic information was “Not Stated”, “Not Known” or “Unresolved” in either source were excluded.
