# Supplementary material for: Understanding the quality of ethnicity data recorded in health-related administrative data sources compared with Census 2021 in England
Source: PLoS Med. 2025 Feb 26;22(2):e1004507. doi: 10.1371/journal.pmed.1004507 (PMC11864522; doi:10.1371/journal.pmed.1004507)
Supplement: S5 Table — (DOCX) [file pmed.1004507.s006.docx]

# **Table S5**. Crosstabulations (A) and level of agreement (B) for 18-category ethnicity coding in individuals in the linked Census 2021-ECIA dataset.

A)

B)

Ethnicity recorded in Census 2021 is reported along the columns and ethnicity recorded in the ECIA is reported along the rows.
Data in panel A are presented as count (n). Data is suppressed if less than 10, and rounded to the nearest 5.
Data in panel B are presented as percentage (%). The Census 2021 ethnic group totals have been used as the denominators when calculating the percentages (%). [c] denotes percentage agreement has not been calculated due to suppression.
The counts and percentages are based on individuals with a stated ethnicity on Census 2021 and the Ethnic Category Information Asset data source.
