# Supplementary material for: Understanding the quality of ethnicity data recorded in health-related administrative data sources compared with Census 2021 in England
Source: PLoS Med. 2025 Feb 26;22(2):e1004507. doi: 10.1371/journal.pmed.1004507 (PMC11864522; doi:10.1371/journal.pmed.1004507)
Supplement: S6 Table — (DOCX) [file pmed.1004507.s007.docx]

# **Table S6**. Crosstabulations (A) and levels of agreement (B) for 18-category ethnicity coding in individuals in the linked Census 2021-GDPPR recency unknown only dataset.

A)

B)

Ethnicity recorded in Census 2021 is reported along the columns and ethnicity recorded in the GDPPR recency unknown only is reported along the rows.
Data in panel A are presented as count (n). Data is suppressed if less than 10, and rounded to the nearest 5.
Data in panel B are presented as percentage (%). The Census 2021 ethnic group totals have been used as the denominators when calculating the percentages (%). [c] denotes percentage agreement has not been calculated due to suppression.
