# Supplementary material for: Understanding the quality of ethnicity data recorded in health-related administrative data sources compared with Census 2021 in England
Source: PLoS Med. 2025 Feb 26;22(2):e1004507. doi: 10.1371/journal.pmed.1004507 (PMC11864522; doi:10.1371/journal.pmed.1004507)
Supplement: S11 Table — (DOCX) [file pmed.1004507.s012.docx]

# **Table S11**. Crosstabulations (A) and level of agreement (B) for 5-category ethnicity coding in individuals in the linked Census 2021-ECIA dataset.

A)

| **Ethnicity recorded in health data source** | **Ethnicity recorded in Census 2021** | | | | |
| --- | --- | --- | --- | --- | --- |
|  | **Asian, Asian British or Asian Welsh** | **Black, Black British, Black Welsh, Caribbean or African** | **Mixed or Multiple ethnic groups** | **White** | **Other ethnic group** |
| **Asian or Asian British** | 3924595 | 18110 | 72560 | 30815 | 259595 |
| **Black or Black British** | 17590 | 1448405 | 87260 | 25010 | 50110 |
| **Mixed** | 100255 | 132620 | 552995 | 136265 | 63025 |
| **White** | 170175 | 101625 | 523390 | 38344435 | 287420 |
| **Other Ethnic Group** | 221745 | 59480 | 90430 | 388375 | 278920 |
| **Not linked** | 312320 | 185390 | 128035 | 3207385 | 101995 |

B)

| **Ethnicity recorded in health data source** | **Ethnicity recorded in Census 2021** | | | | |
| --- | --- | --- | --- | --- | --- |
|  | **Asian, Asian British or Asian Welsh** | **Black, Black British, Black Welsh, Caribbean or African** | **Mixed or Multiple ethnic groups** | **White** | **Other ethnic group** |
| **Asian or Asian British** | 91.1 | 0.4 | 1.7 | 0.7 | 6 |
| **Black or Black British** | 1.1 | 88.9 | 5.4 | 1.5 | 3.1 |
| **Mixed** | 10.2 | 13.5 | 56.1 | 13.8 | 6.4 |
| **White** | 0.4 | 0.3 | 1.3 | 97.3 | 0.7 |
| **Other Ethnic Group** | 21.3 | 5.7 | 8.7 | 37.4 | 26.8 |

Ethnicity recorded in Census 2021 is reported along the columns and ethnicity recorded in the ECIA is reported along the rows.
Data in panel A are presented as count (n). Data is suppressed if less than 10, and rounded to the nearest 5.
Data in panel B are presented as percentage (%). The Census 2021 ethnic group totals have been used as the denominators when calculating the percentages (%). [c] denotes percentage agreement has not been calculated due to suppression.
The counts and percentages are based on individuals with a stated ethnicity on Census 2021 and the Ethnic Category Information Asset data source.
