# Supplementary material for: Understanding the quality of ethnicity data recorded in health-related administrative data sources compared with Census 2021 in England
Source: PLoS Med. 2025 Feb 26;22(2):e1004507. doi: 10.1371/journal.pmed.1004507 (PMC11864522; doi:10.1371/journal.pmed.1004507)
Supplement: S12 Table — (DOCX) [file pmed.1004507.s013.docx]

# **Table S12**. Crosstabulations (A) and level of agreement (B) for 5-category ethnicity coding in individuals in the linked Census 2021-GDPPR recency unknown only dataset.

A)

| **Ethnicity recorded in health data source** | **Ethnicity recorded in Census 2021** | | | | |
| --- | --- | --- | --- | --- | --- |
|  | **Asian, Asian British or Asian Welsh** | **Black, Black British, Black Welsh, Caribbean or African** | **Mixed or Multiple ethnic groups** | **White** | **Other ethnic group** |
| **Asian or Asian British** | 3704165 | 12965 | 61400 | 22435 | 235730 |
| **Black or Black British** | 12575 | 1330230 | 71620 | 19205 | 48140 |
| **Mixed** | 86745 | 133365 | 486900 | 88825 | 56925 |
| **White** | 153800 | 101500 | 463255 | 34180635 | 260170 |
| **Other Ethnic Group** | 141715 | 30105 | 59600 | 234170 | 242125 |
| **Not known** | 305 | 90 | 170 | 5435 | 110 |
| **Not stated** | 89360 | 45295 | 55285 | 921980 | 37835 |
| **Unresolved** | 23450 | 14525 | 12110 | 84565 | 8510 |
| **Not linked** | 534570 | 277550 | 244335 | 6575040 | 151510 |

B)

| **Ethnicity recorded in health data source** | **Ethnicity recorded in Census 2021** | | | | |
| --- | --- | --- | --- | --- | --- |
|  | **Asian, Asian British or Asian Welsh** | **Black, Black British, Black Welsh, Caribbean or African** | **Mixed or Multiple ethnic groups** | **White** | **Other ethnic group** |
| **Asian or Asian British** | 91.8 | 0.3 | 1.5 | 0.6 | 5.8 |
| **Black or Black British** | 0.8 | 89.8 | 4.8 | 1.3 | 3.2 |
| **Mixed** | 10.2 | 15.6 | 57.1 | 10.4 | 6.7 |
| **White** | 0.4 | 0.3 | 1.3 | 97.2 | 0.7 |
| **Other Ethnic Group** | 20 | 4.3 | 8.4 | 33.1 | 34.2 |

Ethnicity recorded in Census 2021 is reported along the columns and ethnicity recorded in the GDPPR recency unknown only is reported along the rows.
Data in panel A are presented as count (n). Data is suppressed if less than 10, and rounded to the nearest 5.
Data in panel B are presented as percentage (%). The Census 2021 ethnic group totals have been used as the denominators when calculating the percentages (%). [c] denotes percentage agreement has not been calculated due to suppression.
The counts are based on individuals with a stated ethnicity on Census 2021 and the General Practice Extraction Service (GPES) Data for Pandemic Planning and Research (GDPPR) data source.
