# Supplementary material for: Understanding the quality of ethnicity data recorded in health-related administrative data sources compared with Census 2021 in England
Source: PLoS Med. 2025 Feb 26;22(2):e1004507. doi: 10.1371/journal.pmed.1004507 (PMC11864522; doi:10.1371/journal.pmed.1004507)
Supplement: S13 Table — (DOCX) [file pmed.1004507.s014.docx]

# **Table S13**. Crosstabulations (A) and level of agreement (B) for 5-category ethnicity coding in individuals in the linked Census 2021-GDPPR modal unknown only dataset.

A)

| **Ethnicity recorded in health data source** | **Ethnicity recorded in Census 2021** | | | | |
| --- | --- | --- | --- | --- | --- |
|  | **Asian, Asian British or Asian Welsh** | **Black, Black British, Black Welsh, Caribbean or African** | **Mixed or Multiple ethnic groups** | **White** | **Other ethnic group** |
| **Asian or Asian British** | 3558680 | 8995 | 46850 | 15570 | 218775 |
| **Black or Black British** | 7445 | 1218520 | 51495 | 11890 | 42615 |
| **Mixed** | 49145 | 85755 | 393535 | 55200 | 40515 |
| **White** | 99370 | 73005 | 400405 | 33008280 | 204605 |
| **Other Ethnic Group** | 83855 | 20965 | 44940 | 176465 | 209515 |
| **Not known** | 305 | 90 | 170 | 5435 | 110 |
| **Not stated** | 68080 | 37560 | 47430 | 793615 | 29865 |
| **Unresolved** | 345230 | 223190 | 225520 | 1490790 | 143550 |
| **Not linked** | 534570 | 277550 | 244335 | 6575040 | 151510 |

B)

| **Ethnicity recorded in health data source** | **Ethnicity recorded in Census 2021** | | | | |
| --- | --- | --- | --- | --- | --- |
|  | **Asian, Asian British or Asian Welsh** | **Black, Black British, Black Welsh, Caribbean or African** | **Mixed or Multiple ethnic groups** | **White** | **Other ethnic group** |
| **Asian or Asian British** | 92.5 | 0.2 | 1.2 | 0.4 | 5.7 |
| **Black or Black British** | 0.6 | 91.5 | 3.9 | 0.9 | 3.2 |
| **Mixed** | 7.9 | 13.7 | 63.1 | 8.8 | 6.5 |
| **White** | 0.3 | 0.2 | 1.2 | 97.7 | 0.6 |
| **Other Ethnic Group** | 15.7 | 3.9 | 8.4 | 32.9 | 39.1 |

Ethnicity recorded in Census 2021 is reported along the columns and ethnicity recorded in the GDPPR modal unknown only is reported along the rows.
Data in panel A are presented as count (n). Data is suppressed if less than 10, and rounded to the nearest 5.
Data in panel B are presented as percentage (%). The Census 2021 ethnic group totals have been used as the denominators when calculating the percentages (%). [c] denotes percentage agreement has not been calculated due to suppression.
The counts are based on individuals with a stated ethnicity on Census 2021 and the General Practice Extraction Service (GPES) Data for Pandemic Planning and Research (GDPPR) data source.
