# Supplementary material for: Understanding the quality of ethnicity data recorded in health-related administrative data sources compared with Census 2021 in England
Source: PLoS Med. 2025 Feb 26;22(2):e1004507. doi: 10.1371/journal.pmed.1004507 (PMC11864522; doi:10.1371/journal.pmed.1004507)
Supplement: S14 Table — (DOCX) [file pmed.1004507.s015.docx]

# **Table S14**. Crosstabulations (A) and level of agreement (B) for 5-category ethnicity coding in individuals in the linked Census 2021-HES recency unknown only dataset.

A)

| **Ethnicity recorded in health data source** | **Ethnicity recorded in Census 2021** | | | | |
| --- | --- | --- | --- | --- | --- |
|  | **Asian, Asian British or Asian Welsh** | **Black, Black British, Black Welsh, Caribbean or African** | **Mixed or Multiple ethnic groups** | **White** | **Other ethnic group** |
| **Asian or Asian British** | 2892110 | 21430 | 61400 | 34420 | 205810 |
| **Black or Black British** | 20035 | 1144090 | 78205 | 29860 | 34285 |
| **Mixed** | 72805 | 77460 | 444550 | 151130 | 41525 |
| **White** | 135080 | 59150 | 413260 | 32298330 | 208680 |
| **Other Ethnic Group** | 283660 | 100875 | 104860 | 538225 | 203455 |
| **Not known** | 170800 | 64575 | 44040 | 1283785 | 38755 |
| **Not stated** | 647135 | 299565 | 204310 | 5092390 | 177005 |
| **Unresolved** | 18740 | 8340 | 6660 | 75855 | 4590 |
| **Not linked** | 506315 | 170145 | 97385 | 2628300 | 126960 |

B)

| **Ethnicity recorded in health data source** | **Ethnicity recorded in Census 2021** | | | | |
| --- | --- | --- | --- | --- | --- |
|  | **Asian, Asian British or Asian Welsh** | **Black, Black British, Black Welsh, Caribbean or African** | **Mixed or Multiple ethnic groups** | **White** | **Other ethnic group** |
| **Asian or Asian British** | 90 | 0.7 | 1.9 | 1.1 | 6.4 |
| **Black or Black British** | 1.5 | 87.6 | 6 | 2.3 | 2.6 |
| **Mixed** | 9.2 | 9.8 | 56.5 | 19.2 | 5.3 |
| **White** | 0.4 | 0.2 | 1.2 | 97.5 | 0.6 |
| **Other Ethnic Group** | 23 | 8.2 | 8.5 | 43.7 | 16.5 |

Ethnicity recorded in Census 2021 is reported along the columns and ethnicity recorded in the HES recency unknown only is reported along the rows.
Data in panel A are presented as count (n). Data is suppressed if less than 10, and rounded to the nearest 5.
Data in panel B are presented as percentage (%). The Census 2021 ethnic group totals have been used as the denominators when calculating the percentages (%). [c] denotes percentage agreement has not been calculated due to suppression.
The counts are based on individuals with a stated ethnicity on Census 2021 and the HES data source.
