# Supplementary material for: Understanding the quality of ethnicity data recorded in health-related administrative data sources compared with Census 2021 in England
Source: PLoS Med. 2025 Feb 26;22(2):e1004507. doi: 10.1371/journal.pmed.1004507 (PMC11864522; doi:10.1371/journal.pmed.1004507)
Supplement: S15 Table — (DOCX) [file pmed.1004507.s016.docx]

# **Table S15**. Crosstabulations (A) and level of agreement (B) for 5-category ethnicity coding in individuals in the linked Census 2021-HES modal unknown only dataset.

A)

| **Ethnicity recorded in health data source** | **Ethnicity recorded in Census 2021** | | | | |
| --- | --- | --- | --- | --- | --- |
|  | **Asian, Asian British or Asian Welsh** | **Black, Black British, Black Welsh, Caribbean or African** | **Mixed or Multiple ethnic groups** | **White** | **Other ethnic group** |
| **Asian or Asian British** | 2961300 | 19000 | 59540 | 30960 | 206555 |
| **Black or Black British** | 17125 | 1180105 | 76930 | 22085 | 34625 |
| **Mixed** | 66460 | 71550 | 446940 | 111845 | 39405 |
| **White** | 122120 | 53095 | 409400 | 32977160 | 210850 |
| **Other Ethnic Group** | 244905 | 79180 | 92565 | 410380 | 201220 |
| **Not known** | 166985 | 63135 | 42855 | 1243395 | 38060 |
| **Not stated** | 524120 | 237810 | 165985 | 3997415 | 147505 |
| **Unresolved** | 137350 | 71610 | 63070 | 710745 | 35885 |
| **Not linked** | 506315 | 170145 | 97385 | 2628300 | 126960 |

B)

| **Ethnicity recorded in health data source** | **Ethnicity recorded in Census 2021** | | | | |
| --- | --- | --- | --- | --- | --- |
|  | **Asian, Asian British or Asian Welsh** | **Black, Black British, Black Welsh, Caribbean or African** | **Mixed or Multiple ethnic groups** | **White** | **Other ethnic group** |
| **Asian or Asian British** | 90.4 | 0.6 | 1.8 | 0.9 | 6.3 |
| **Black or Black British** | 1.3 | 88.7 | 5.8 | 1.7 | 2.6 |
| **Mixed** | 9 | 9.7 | 60.7 | 15.2 | 5.4 |
| **White** | 0.4 | 0.2 | 1.2 | 97.6 | 0.6 |
| **Other Ethnic Group** | 23.8 | 7.7 | 9 | 39.9 | 19.6 |

Ethnicity recorded in Census 2021 is reported along the columns and ethnicity recorded in the HES modal unknown only is reported along the rows.
Data in panel A are presented as count (n). Data is suppressed if less than 10, and rounded to the nearest 5.
Data in panel B are presented as percentage (%). The Census 2021 ethnic group totals have been used as the denominators when calculating the percentages (%). [c] denotes percentage agreement has not been calculated due to suppression.
The counts are based on individuals with a stated ethnicity on Census 2021 and the HES data source.
