# Supplementary material for: Understanding the quality of ethnicity data recorded in health-related administrative data sources compared with Census 2021 in England
Source: PLoS Med. 2025 Feb 26;22(2):e1004507. doi: 10.1371/journal.pmed.1004507 (PMC11864522; doi:10.1371/journal.pmed.1004507)
Supplement: S16 Table — (DOCX) [file pmed.1004507.s017.docx]

# **Table S16**. Crosstabulations (A) and level of agreement (B) for 5-category ethnicity coding in individuals in the linked Census 2021-TT recency unknown only dataset.

A)

| **Ethnicity recorded in health data source** | **Ethnicity recorded in Census 2021** | | | | |
| --- | --- | --- | --- | --- | --- |
|  | **Asian, Asian British or Asian Welsh** | **Black, Black British, Black Welsh, Caribbean or African** | **Mixed or Multiple ethnic groups** | **White** | **Other ethnic group** |
| **Asian or Asian British** | 287045 | 930 | 4680 | 2305 | 21760 |
| **Black or Black British** | 735 | 142050 | 7115 | 1870 | 3415 |
| **Mixed** | 6890 | 7355 | 93245 | 16340 | 6885 |
| **White** | 6650 | 4580 | 33800 | 4695780 | 23545 |
| **Other Ethnic Group** | 13475 | 4355 | 5620 | 23165 | 29435 |
| **Not stated** | 22495 | 12075 | 11505 | 317210 | 9020 |
| **Not known** | 28880 | 13995 | 11300 | 358115 | 10115 |
| **Unresolved** | 1140 | 540 | 550 | 12480 | 445 |
| **Value outside of national code** | 400 | 125 | 145 | 6985 | 185 |
| **Data not recorded** | 5080 | 2515 | 2040 | 60490 | 1645 |
| **Not linked** | 4373895 | 1757115 | 1284670 | 36637550 | 934610 |

B)

| **Ethnicity recorded in health data source** | **Ethnicity recorded in Census 2021** | | | | |
| --- | --- | --- | --- | --- | --- |
|  | **Asian, Asian British or Asian Welsh** | **Black, Black British, Black Welsh, Caribbean or African** | **Mixed or Multiple ethnic groups** | **White** | **Other ethnic group** |
| **Asian or Asian British** | 90.6 | 0.3 | 1.5 | 0.7 | 6.9 |
| **Black or Black British** | 0.5 | 91.5 | 4.6 | 1.2 | 2.2 |
| **Mixed** | 5.3 | 5.6 | 71.3 | 12.5 | 5.3 |
| **White** | 0.1 | 0.1 | 0.7 | 98.6 | 0.5 |
| **Other Ethnic Group** | 17.7 | 5.7 | 7.4 | 30.5 | 38.7 |

Ethnicity recorded in Census 2021 is reported along the columns and ethnicity recorded in the NHS Talking Therapies recency unknown only is reported along the rows.
Data in panel A are presented as count (n). Data is suppressed if less than 10, and rounded to the nearest 5.
Data in panel B are presented as percentage (%). The Census 2021 ethnic group totals have been used as the denominators when calculating the percentages (%). [c] denotes percentage agreement has not been calculated due to suppression.
The counts are based on individuals with a stated ethnicity on Census 2021 and the NHS Talking Therapies data source.
