# Supplementary material for: Understanding the quality of ethnicity data recorded in health-related administrative data sources compared with Census 2021 in England
Source: PLoS Med. 2025 Feb 26;22(2):e1004507. doi: 10.1371/journal.pmed.1004507 (PMC11864522; doi:10.1371/journal.pmed.1004507)
Supplement: S17 Table — (DOCX) [file pmed.1004507.s018.docx]

# **Table S17.** Percentage of agreement between health datasets and Census 2021 using 18-category ethnicities in a sensitivity analysis restricting the population to people with a stated ethnic category in each of Census 2021, GDPPR and HES data sources only, England.

| **Ethnicity** | **GDPPR modal, Unknown Only (%)** | **GDPPR recency, Unknown Only (%)** | **HES modal, Unknown Only (%)** | **HES recency, Unknown Only (%)** |
| --- | --- | --- | --- | --- |
| **White British** | 97.0 | 96.9 | 97.0 | 96.9 |
| **Bangladeshi** | 97.0 | 96.8 | 94.4 | 94.0 |
| **Pakistani** | 91.8 | 91.6 | 88.3 | 88.1 |
| **Chinese** | 91.2 | 90.9 | 82.2 | 81.6 |
| **Indian** | 88.2 | 88.0 | 83.9 | 83.6 |
| **Black African** | 83.6 | 83.3 | 78.8 | 77.6 |
| **Black Caribbean** | 79.0 | 78.1 | 72.2 | 71.5 |
| **Arab** | 75.3 | 72.6 | [x] | [x] |
| **White Irish** | 72.2 | 67.7 | 59.7 | 57.5 |
| **Mixed White and Black Caribbean** | 70.2 | 68.4 | 69.6 | 68.8 |
| **Mixed White and Asian** | 62.6 | 60.3 | 55.4 | 54.1 |
| **Other Asian** | 55.3 | 51.2 | 36.2 | 34.4 |
| **Other White** | 52.7 | 46.4 | 53.2 | 51.6 |
| **Mixed White and Black African** | 44.9 | 40.4 | 46.6 | 45.3 |
| **Other Mixed** | 25.8 | 23.9 | 17.0 | 14.6 |
| **Any Other Ethnic Group** | 24.8 | 19.4 | 10.9 | 9.6 |
| **Other Black** | 18.5 | 15.2 | 12.1 | 12.0 |
| **Traveller** | 7.1 | 6.4 | [x] | [x] |
| **Roma** | [x] | [x] | [x] | [x] |

Agreement is based on linked individuals with a stated ethnicity in the relevant health dataset and Census 2021. “Not Stated”, “Not Known” or “Unresolved” categories were excluded from the agreement calculation. The population included is therefore different for each data source.
For each source, the health data ethnic group totals have been used as denominators when calculating percentages.
The Arab and Traveller ethnic group categories are not available in HES, so agreement for these categories are only presented for ECIA and GDPPR. The Roma ethnic group is not available for any dataset. This is denoted in the table as [x].
