# Supplementary material for: Understanding the quality of ethnicity data recorded in health-related administrative data sources compared with Census 2021 in England
Source: PLoS Med. 2025 Feb 26;22(2):e1004507. doi: 10.1371/journal.pmed.1004507 (PMC11864522; doi:10.1371/journal.pmed.1004507)
Supplement: S18 Table — (DOCX) [file pmed.1004507.s019.docx]

# **Table S18.** Percentage of agreement between health datasets and Census 2021 using 5-category ethnicities in a sensitivity analysis restricting the population to people with a stated ethnic category in each of Census 2021, GDPPR and HES data sources only, England.

| **Ethnicity** | **GDPPR modal, Unknown Only (%)** | **GDPPR recency, Unknown Only (%)** | **HES modal, Unknown Only (%)** | **HES recency, Unknown Only (%)** |
| --- | --- | --- | --- | --- |
| **White** | 97.9 | 97.8 | 98.0 | 98.0 |
| **Asian** | 92.6 | 92.6 | 91.5 | 91.3 |
| **Black** | 91.7 | 91.0 | 89.3 | 88.7 |
| **Mixed or Multiple ethnic groups** | 65.3 | 61.4 | 58.5 | 54.3 |
| **Other ethnic group** | 38.5 | 33.6 | 17.5 | 15.3 |

Agreement is based on linked individuals with a stated ethnicity in the relevant health dataset and Census 2021. “Not Stated”, “Not Known” or “Unresolved” categories were excluded from the agreement calculation. The population included is therefore different for each data source.
For each source, the health data ethnic group totals have been used as denominators when calculating percentages.
HES and TT data does not include ethnic categories for Traveller or Arab groups, therefore they cannot be included overall agreement total (numerator). However, as Census 2021 does include Traveller and Arab ethnic categories, they are included in the total count for all ethnic categories (denominator). This methodology has been used in previous ONS publications.
The Chinese category has been included in the 'Asian/Asian British' category, in line with the Government Statistical Service Harmonised Standard.
