# Supplementary material for: Understanding the quality of ethnicity data recorded in health-related administrative data sources compared with Census 2021 in England
Source: PLoS Med. 2025 Feb 26;22(2):e1004507. doi: 10.1371/journal.pmed.1004507 (PMC11864522; doi:10.1371/journal.pmed.1004507)
Supplement: S19 Table — (DOCX) [file pmed.1004507.s020.docx]

# **Table S19**. Comparison of sensitivity and positive predictive value in 18-category ethnicity categories within Census 2021 to the ECIA, GDPPR, HES and TT data sources, England.

|  |  | **Bangladeshi** | **Chinese** | **Indian** | **Pakistani** | **Other Asian** | **Black African** | **Black Caribbean** | **Other Black** | **White and Asian** | **White and Black African** | **White and Black Caribbean** | **Other Mixed** | **White British** | **Gypsy or Irish Traveller** | **White Irish** | **Roma** | **Other White** | **Any other ethnic group** | **Arab** |
| --- | --- | --- | --- | --- | --- | --- | --- | --- | --- | --- | --- | --- | --- | --- | --- | --- | --- | --- | --- | --- |
| **Census 2021 – ECIA** | **Sensitivity**  **(%)** | 83.6 | 77.7 | 81.3 | 85.5 | 53.8 | 68.3 | 58.8 | 22.4 | 30.2 | 31.4 | 36.1 | 22.1 | 94.6 | 1.0 | 42.7 | **-** | 75.7 | 22.1 | 5.6 |
|  | **PPV (%)** | 95.4 | 88.2 | 86.8 | 89.7 | 45.8 | 81.2 | 74.1 | 13.8 | 56.0 | 37.9 | 65.7 | 19.5 | 96.2 | 5.3 | 64.6 | **-** | 50.3 | 15.1 | 74.5 |
| **Census 2021 – GDPPR-recency** | **Sensitivity**  **(%)** | 87.1 | 79.4 | 84.3 | 88.5 | 58.0 | 70.7 | 58.8 | 23.4 | 31.6 | 32.9 | 37.7 | 23.1 | 94.3 | 2.9 | 46.2 | **-** | 78.8 | 20.4 | 11.6 |
|  | **PPV (%)** | 96.1 | 90.7 | 88.0 | 90.9 | 50.1 | 82.9 | 76.3 | 14.5 | 56.3 | 35.3 | 64.6 | 22.5 | 96.2 | 0.1 | 66.4 | **-** | 49.8 | 19.1 | 73.7 |
| **Census 2021 – GDPPR-modal** | **Sensitivity**  **(%)** | 90.9 | 85.5 | 89.1 | 92.1 | 65.9 | 81.2 | 66.0 | 19.7 | 32.2 | 33.3 | 38.1 | 22.4 | 95.9 | 2.5 | 46.0 | **-** | 82.8 | 22.0 | 21.1 |
|  | **PPV (%)** | 96.9 | 91.9 | 89.1 | 91.8 | 57.1 | 84.4 | 78.8 | 17.9 | 60.9 | 41.9 | 68.7 | 25.8 | 96.8 | 0.1 | 73.3 | **-** | 57.7 | 24.6 | 76.8 |
| **Census 2021 – HES-recency** | **Sensitivity**  **(%)** | 74.7 | 71.1 | 71.5 | 78.8 | 44.5 | 64.3 | 57.0 | 19.2 | 27.1 | 28.6 | 34.1 | 19.7 | 95.2 | **-** | 33.1 | **-** | 67.4 | 23.9 | **-** |
|  | **PPV (%)** | 93.3 | 79.5 | 82.5 | 86.9 | 33.9 | 75.6 | 69.9 | 12.6 | 55.2 | 46.4 | 68.4 | 15.5 | 96.4 | **-** | 57.3 | **-** | 49.5 | 10.1 | **-** |
| **Census 2021 – HES-modal** | **Sensitivity**  **(%)** | 77.6 | 73.8 | 75.0 | 81.3 | 46.1 | 67.8 | 60.8 | 19.0 | 28.0 | 29.9 | 35.2 | 20.1 | 96.0 | **-** | 33.5 | **-** | 69.5 | 23.2 | **-** |
|  | **PPV (%)** | 94.0 | 80.8 | 83.0 | 87.4 | 36.4 | 77.5 | 71.3 | 12.8 | 56.8 | 48.3 | 69.6 | 18.0 | 96.5 | **-** | 60.1 | **-** | 51.5 | 11.8 | **-** |
| **Census 2021 – NHS Talking Therapies-recency** | **Sensitivity**  **(%)** | 88.6 | 71.8 | 87.5 | 87.5 | 57.0 | 78.2 | 76.6 | 22.1 | 49.8 | 49.0 | 66.1 | 38.9 | 98.2 | **-** | 55.2 | **-** | 73.8 | 28.9 | **-** |
|  | **PPV (%)** | 97.1 | 84.9 | 85.5 | 92.5 | 47.5 | 86.1 | 76.8 | 19.2 | 67.7 | 63.9 | 74.8 | 34.4 | 97.4 | **-** | 70.6 | **-** | 70.9 | 24.8 | **-** |

HES and TT data does not include ethnic categories for Traveller or Arab groups, therefore no sensitivity or positive predictive value has been calculated.
