# Supplementary material for: Understanding the quality of ethnicity data recorded in health-related administrative data sources compared with Census 2021 in England
Source: PLoS Med. 2025 Feb 26;22(2):e1004507. doi: 10.1371/journal.pmed.1004507 (PMC11864522; doi:10.1371/journal.pmed.1004507)
Supplement: S20 Table — (DOCX) [file pmed.1004507.s021.docx]

# **Table S20**. Count of people in the linked datasets created to compare consistency of ethnicity recording in health sources with the Census 2021, England.
