# Supplementary material for: Understanding the quality of ethnicity data recorded in health-related administrative data sources compared with Census 2021 in England
Source: PLoS Med. 2025 Feb 26;22(2):e1004507. doi: 10.1371/journal.pmed.1004507 (PMC11864522; doi:10.1371/journal.pmed.1004507)
Supplement: S1 Fig — (DOCX) [file pmed.1004507.s022.docx]

# **Figure S1.** Percentage of agreement between health datasets and Census 2021 using 5-category ethnicities, England.


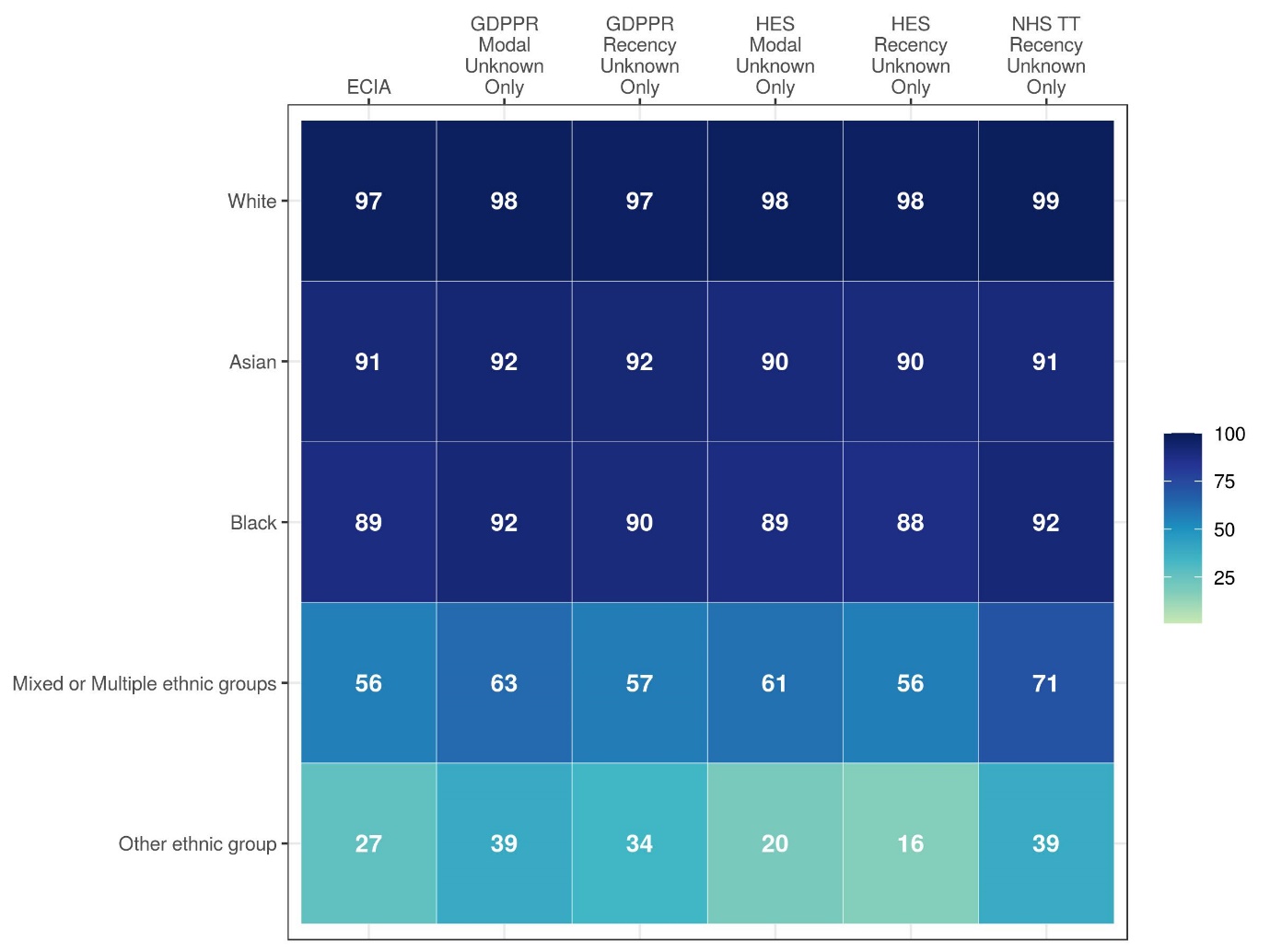


Data presented is percentage (%).
Agreement is based on linked individuals with a stated ethnicity in the relevant health dataset and Census 2021. “Not Stated”, “Not Known” or “Unresolved” categories were excluded from the agreement calculation. The population included is therefore different for each data source.
For each source, the health data ethnic group totals have been used as denominators when calculating percentages.
HES and TT data does not include ethnic categories for Traveller or Arab groups, therefore they cannot be included overall agreement total (numerator). However, as Census 2021 does include Traveller and Arab ethnic categories, they are included in the total count for all ethnic categories (denominator). This methodology has been used in previous ONS publications.
The Chinese category has been included in the 'Asian/Asian British' category, in line with the Government Statistical Service Harmonised Standard.
For GDPPR, HES and TT data sources, these data refer to when the Unknown only reallocation methodology has been applied.
