# Supplementary material for: Understanding the quality of ethnicity data recorded in health-related administrative data sources compared with Census 2021 in England
Source: PLoS Med. 2025 Feb 26;22(2):e1004507. doi: 10.1371/journal.pmed.1004507 (PMC11864522; doi:10.1371/journal.pmed.1004507)
Supplement: S2 Fig — (DOCX) [file pmed.1004507.s023.docx]

# **Figure S2**. Percentage of agreement between health datasets and Census 2021 using 18-category ethnicities in a sensitivity analysis restricting the back series of data to 1 April 2015, England.


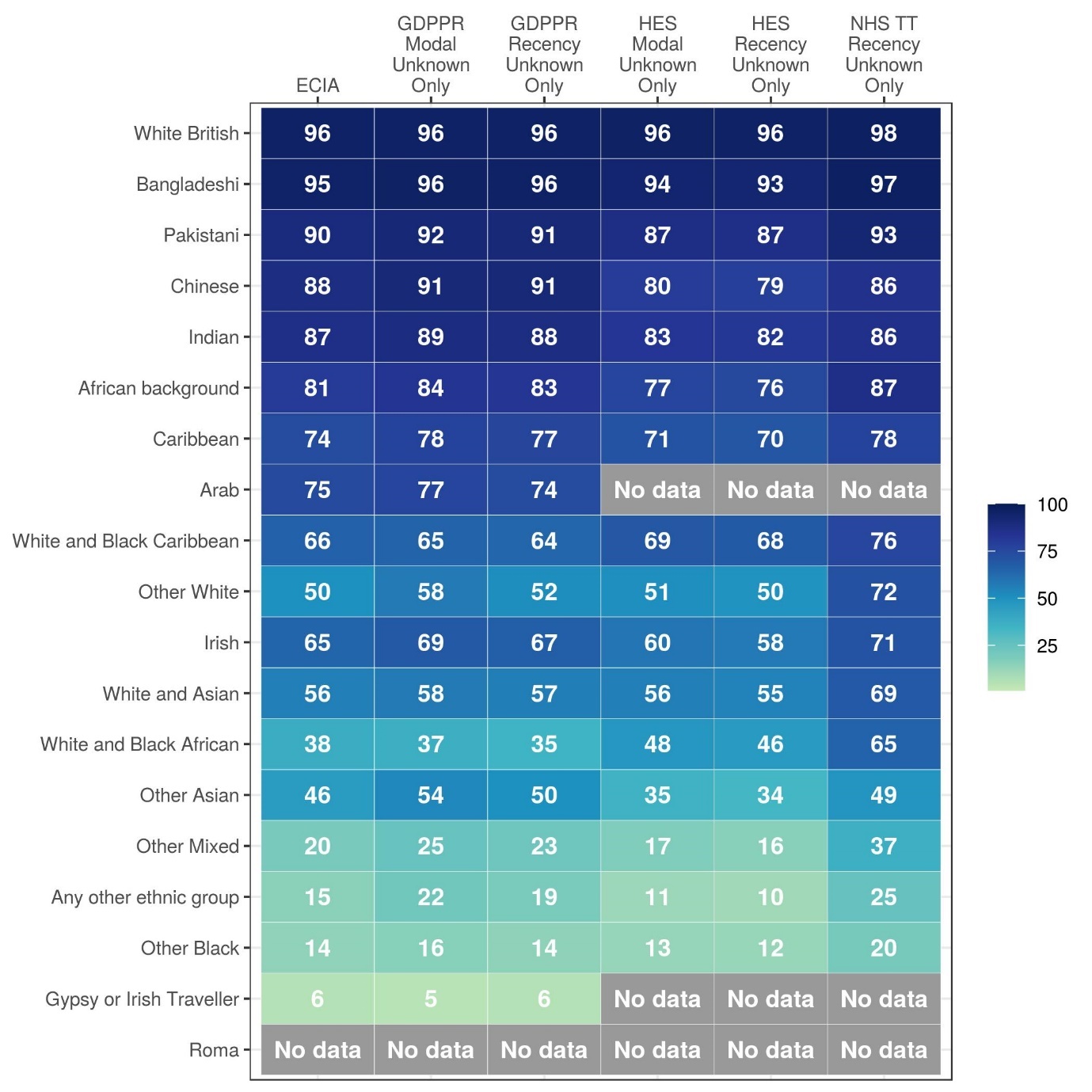


Data are presented as percentage (%).
Agreement is based on linked individuals with a stated ethnicity in the relevant health dataset and Census 2021. “Not Stated”, “Not Known” or “Unresolved” categories were excluded from the agreement calculation. The population included is therefore different for each data source.
For each source, the health data ethnic group totals have been used as denominators when calculating percentages.
The Arab and Traveller ethnic group categories are not available in HES or NHS TT, so agreement for these categories are only presented for ECIA and GDPPR. The Roma ethnic group is not available for any dataset.
For GDPPR, HES and TT data sources, these data refer to when the Unknown only reallocation methodology has been applied.
